# Non-Lethal Detection of *Frog Virus 3*-Like (RUK13) and *Common Midwife Toad Virus*-Like (PDE18) Ranaviruses in Two UK-Native Amphibian Species

**DOI:** 10.3390/v14122635

**Published:** 2022-11-25

**Authors:** Charlotte E. Ford, Lola M. Brookes, Emily Skelly, Chris Sergeant, Tresai Jordine, Francois Balloux, Richard A. Nichols, Trenton W. J. Garner

**Affiliations:** 1 School of Biological and Behavioural Sciences, Queen Mary University of London, Mile End Road, London E1 4NS, UK; 2 Zoological Society of London, Institute of Zoology, Nuffield Building, Outer Circle, London NW8 7LS, UK; 3 UCL Genetics Institute, University College London, Gower Street, London WC1E 6BT, UK; 4 RVC Animal Welfare Science and Ethics, The Royal Veterinary College, Hawkshead Lane, Hatfield AL9 7TA, UK

**Keywords:** non-lethal detection, buccal swab, FV3, CMTV, common frog, common toad

## Abstract

Ranaviruses have been involved in amphibian mass mortality events worldwide. Effective screening to control this pathogen is essential; however, current sampling methods are unsuitable for the detection of subclinical infections. Non-lethal screening is needed to prevent both further spread of ranavirus and losses of at-risk species. To assess non-lethal sampling methods, we conducted two experiments: bath exposing common frogs to RUK13 ranavirus at three concentrations, and exposing common toads to RUK13 or PDE18. Non-lethal sampling included buccal, digit, body and tank swabs, along with toe clips and stool taken across three time-points post-exposure. The presence/load of ranavirus was examined using quantitative PCR in 11 different tissues obtained from the same euthanised animals (incl. liver, gastro-intestinal tract and kidney). Buccal swab screening had the highest virus detection rate in both species (62% frogs; 71% toads) and produced consistently high virus levels compared to other non-lethal assays. The buccal swab was effective across multiple stages of infection and differing infection intensities, though low levels of infection were more difficult to detect. Buccal swab assays competed with, and even outperformed, lethal sampling in frogs and toads, respectively. Successful virus detection in the absence of clinical signs was observed (33% frogs; 50% toads); we found no difference in detectability for RUK13 and PDE18. Our results suggest that buccal swabbing could replace lethal sampling for screening and be introduced as standard practice for ranavirus surveillance.

## 1. Introduction

The genus *Ranavirus* contains a group of double-stranded DNA viruses capable of infecting a broad host range spanning amphibians, reptiles and fish [1]. Whilst ranaviruses can cause a disease known as ranavirosis, these viruses have also been detected in hosts which exhibit no apparent signs of disease; these cases are referred to as subclinical infections, which have been reported in wild amphibians, reptiles and fish and have also been produced experimentally [2,3,4]. Current knowledge of ranavirus presence in the UK is based primarily on submissions of mortality samples to the Garden Wildlife Health project [5]. Non-lethal sampling could provide a more comprehensive picture of ranavirus in the UK landscape, both in terms of prevalence and presence of different ranavirus species.

The World Organisation for Animal Health (OIE) has classified ranaviruses as notifiable pathogens and recommend that, when screening for both clinical (systemic) and sub-clinical infections, that tissue samples be taken [6]. Currently, no suitable sampling strategies have been identified and validated for the detection of ranavirus in animals with no clinical signs of ill health, apart from lethal sampling [7,8,9]. Given that amphibians are currently experiencing global population declines, non-lethal sampling would provide a much needed tool to demonstrate that individuals, which had come under suspicion, were indeed infected [10]. Any reduction in the culling of otherwise healthy individuals would have ethical value, and be of particular importance in species of conservation concern and those that are sold through the pet trade. Non-lethal sampling would have several uses: it could help to improve welfare standards in experiments, provide more widespread surveillance of ranaviruses, and be used to reduce the risk of importation of ranaviruses to new areas via the trade of animals with cryptic infections.

Efforts have been made to identify non-lethal sampling methods that could be used for the screening of ranavirus, but the findings have been inconsistent. Toe clipping, for instance, was found to yield similar results to liver tissue (*Rana clamitans*) and was determined to be better suited for virus surveillance, based on higher viral loads than liver or swabs (*Rana temporaria*). However, it has also been reported, alongside non-lethal sampling in general, to only stand a moderate chance at detecting low grade infections in *R. catebeianus* [9,11,12]. The presence of ranavirus in stool has, so far, only been investigated in one amphibian species, *Lissotriton vulgaris*; it was found to be intermittently present in the faeces [13]. We found only one previous example of buccal swabs for ranavirus detection; a study which focused on two turtle species, and combined the swabbing of the oral cavity and cloaca [14]. In this case, oral-cloacal swabbing yielded more false-negatives than tail clipping. It is unknown how this sampling method, or buccal swabs alone, would perform in amphibians. Several of these studies have argued that tissue sampling is more appropriate for ranavirus monitoring. However, these tissue sampling studies are mostly investigations of mortality or morbidity cases and, consequently, involve hosts that were susceptible to viral disease [5,15]. In ranavirus literature, the most common sample used for ranavirus screening is the liver. This choice coincides with the recommendation of the World Organisation for Animal Health (OIE) that, when sampling amphibians greater than 60 mm in length, the liver, kidney and spleen should be isolated for testing, with the lung and skin being retained as an option if needed [15,16].

There is still uncertainty as to which tissues are targeted at the start of a ranavirus infection and whether the location of the virus changes as an infection progresses; experimental evidence has shown both species and lineage differences [17]. For example, *Common midwife toad virus* (CMTV)-type ranavirus infections in *L. vulgaris* can be first detected in the oral cavity and upper respiratory mucosa, with infections spreading to the connective tissues and vasculature of the gastrointestinal tract. This is followed by widespread disease in organs including skin, kidneys and gonads [13], whereas in *R. sylvatica* exposed to *Frog virus 3* (FV3)-like ranavirus, the skin and bone marrow were the first targets, with slow viral replication observed in the oral, gastrointestinal and pulmonary epithelia. The skin, oral, gastrointestinal, and renal tubular epithelium all appeared to be important sites for FV3 replication and shedding [18]. In the UK, our understanding of ranavirus pathology is limited to end-stage infections. Investigations have found ranavirus within the skin, as well as a wide range of organs in the common frog [19,20]. In order to better screen for ranavirus, we argue that a more thorough understanding of infection dynamics and the relationship across tissues is needed to determine how suited non-lethal sampling is for ranavirus screening.

There are currently two ranavirus amphibian lineages circulating in Europe; the CMTV and FV3 lineages. In continental Europe, ranavirus-induced mortality is predominantly caused by CMTV strains and have resulted in local persistent population declines through outbreaks spanning entire amphibian community assemblages and life stages [21,22,23]. In the UK, however, the opposite is true; FV3-like viruses have been responsible for the population declines of over 80% in common frogs [24,25]. The divergence in prevalence of ranavirus between mainland Europe and the UK may indicate that infection dynamics vary between ranavirus lineages. Better understanding of these dynamics across lineages would help to inform how detectability might vary between ranaviruses.

In this study, we aimed to assess the ability of several non-lethal sampling methods at detecting ranavirus as infection progressed, as well as in hosts with differing infection intensities. We targeted post-metamorphic *R. temporaria* and adult *Bufo bufo* for exposure in two experiments: experiment (1) non-lethal sampling was compared to lethal sampling of various tissue samples. Detectability was assessed across different infection intensities of a single FV3-like ranavirus isolate as well as progression of infection over time, and experiment (2) compared non-lethal sampling methods identified in experiment 1 with a smaller selection of tissue samples (again guided by the previous experiment) for the detection of two ranavirus lineages circulating across Europe.

## 2. Materials and Methods

### 2.1. Biosecurity

Egg clutches brought in from wild sites were kept in the same tanks until metamorphosis. *Rana temporaria* and *Bufo bufo* were housed in separate rooms which contained separate equipment. All liquid waste was treated with Virkon (0.5%) overnight before disposal; all other remaining waste was incinerated. Biosecurity was implemented throughout the experiments, with nitrile gloves changed between treatment group and/or animal handled. The experimental room and equipment used were cleaned with Virkon (0.5%) and Distel (10%) daily. PPE including lab coats and gloves were worn at all times and foot baths containing Virkon were used to ensure no virus escaped the experimental room. Tissue harvesting equipment was treated with Virkon (40%) for 20 min before rinsing with ethanol and double distilled Water (ddH20). All liquid waste was treated with Virkon (0.5%) overnight before disposal, the remaining waste incinerated.

### 2.2. Collection and Rearing

Eighty-four newly metamorphosed *R. temporaria*, collected as spawn in 2019 and transported in fish bags by public transport (under license and with land-owner’s permission) from Palmers Green, London, were used for experiment 1. Fifty-one adult *B. bufo*, collected as spawn in 2017 (under license and with the landowner’s permission) transported in fish bags via car from several locations in/around the Isle of Skye, Scotland, for the second experiment. This species was raised in captivity at the Zoological Society of London, experiencing one period of over-wintering prior to the experiment. Whilst the historic ranavirus-status of these sites are unknown, the site at Palmers Green has a historic ranavirus-free status.

Both spawn and emerging tadpoles were housed in tanks (90 L) with an air pump driven sponge filter, 100% dechlorinated water and containing *Elodea densa*. Tanks were exposed to the elements, allowing natural climatic conditions, but contained within roof-top domiciles to restrict access to licensed personnel only. Spawn were left alone until hatching began, with clutch remnants and underdeveloped/hatched embryos removed. Water changes (approx. 30%) occurred twice weekly, with carcasses and debris removed. Water testing was undertaken using API^®^ test kits (API Lab Testing Limited, Hong Kong, China) to ensure ammonium, nitrite and nitrate levels were low and stable. Tadpoles were fed a combination of algae wafers and spirulina wafers (Aquadip) as well as cubes of inhouse made food (comprised of fish flakes (10 parts), trout pellets (8), grass pellets (8), cuttlefish bone (3), Tubifex (1–2), river shrimps (1–2) and Spirulina algae (2–3)) three times a week. Once all four limbs had emerged from individuals of both species, metamorphs were transferred to semi-aquatic tanks (exo terra faunariums, consisting of a large roof tile, partly submerged in dechlorinated water), until tails had disappeared, and were then moved to terrestrial tanks lined with non-toxic paper toweling. Additionally provided were one Exo Terra reptile hide and one piece of cork-bark for refuge, moss and two Petri dishes filled with dechlorinated water. Stocking densities were set at a maximum of 20 post-metamorphs/adults per terrestrial tank, though tanks numbers fluctuated during the metamorph period as amphibians were sorted by size to assist welfare checks. Amphibians were fed Nutrobal-dusted hatchling brown crickets (post-metamorph frogs) and 2nd brown crickets (adult toads) three times a week.

*R. temporaria* and *B. bufo* were then habituated to climate-controlled animal rooms, after 5 and 6 months of being housed outside, respectively. Exo Terra housing units became soil-based, still with access to two hides and two sources of water. To assist with moisture control, each housing unit was lightly sprayed with dechlorinated water once a week and all boxes pre-experiment, were housed under UVB-lighting. Daily checks were performed to assess tank conditions and health of the animals by visual appearance. Tanks were cleaned twice weekly (substrate changed every 6 weeks), and during this time, a more thorough examination of the animals was performed via handling looking for indications of poor body condition or unusual behaviour.

### 2.3. Experimental Preparation

Individually housed *R. temporaria* and *B. bufo* were acclimatized to experimental conditions for 12 and 7 days prior to exposure, respectively. Housing consisted of a hide and moist, non-toxic paper toweling substrate, which was changed every 4 days and checked daily for standing water, a factor shown to cause irritation to the skin of frogs [12]. Any standing water was either poured away as wastewater or removed by toweling dry the area of the tank affected. Lighting was available for 13 h (frogs) or 8 h (toads) each day and temperature was set to 20 °C, suitable for host-pathogen interactions [26]. At the start of the acclimation period, the frogs were fed 8 Nutrobal-dusted hatchling brown crickets every 3 days, but changed to larger crickets (first instar) after the initial feeding day. Toads continued to be fed 8 Nutrobal-dusted 2nd brown crickets every 4 days. After animals had been randomly allocated to housing, each animal was again randomly assigned to 1 of 4 (experiment 1, *R. temporaria*) or 1 of 3 (experiment 2, *B. bufo*) treatment groups and weighed using scales in grams (two decimal places).

The solution for the control “sham” group, consisted of growth media (500 mL Minimum Essential Medium Eagle (MEM), 10% sterile filtered Fetal Bovine Serum (FBS; Thermofisher (cat. No 10270-106)), 1% L-Glutamine, 1% non-essential amino acids). For experiment 1, frozen RUK13 (*Frog Virus 3*-like ranavirus) [27] stock was diluted to the required concentrations prior to exposure (using the same growth media): 3.06 × 10^8^ TCID_50_/mL (High dose), 3.06 × 10^6.5^ TCID_50_/mL (Medium dose) and 3.06 × 10^5^ TCID_50_/mL (Low dose). For experiment 2, RUK13 and PDE18 (*Common midwife toad virus*-like ranavirus) [12] ranaviruses were grown using epithelioma papulosum cyprinid (EPC) cell lines and growth media (as above). EPC cells were grown in 5% CO_2_ incubators at 28 °C and transferred to new flasks every 5 days. Cells were inoculated with either RUK13 or PDE18 and incubated at 24 °C (RUK13) and 20 °C (PDE18) for 5 days (optimal temperatures for growth of each ranavirus) [26]. Ranavirus was isolated using a combination of freeze–thaw (3×) and centrifugation (5 min at 500× *g*) and ranavirus titre was determined using the Median Tissue Culture Infectious Dose (TCID_50_) assay. PDE18 (1.25 × 10^8^ TCID_50_/mL) and RUK13 (3.16 × 10^7^ TCID_50_/mL) were then diluted to 6.12 × 10^5.5^ TCID_50_/mL using growth media.

### 2.4. Experiment 1: Detectability of RUK13 in Post-Metamorphic R. temporaria

**Figure 1:**
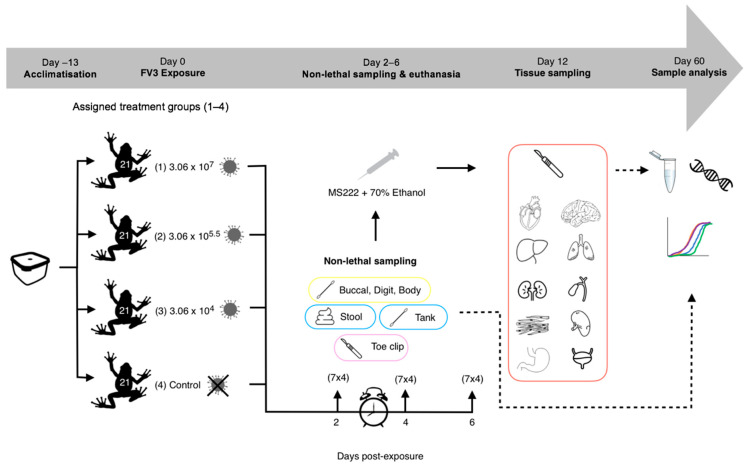
Experimental 1 workflow (experimental design and post-experimental processing).

*R. temporaria* were transferred from housing to Petri dishes (6 cm × 1.5 cm) containing dechlorinated water (9 mL), according to treatment (Figure 1). 1 mL of ranavirus/sham mix was added to each Petri dish (approx. 6 frogs/minute/treatment) to make the final concentrations 3.06 × 10^7^ TCID_50_/mL (High dose), 3.06 × 10^5.5^ TCID_50_/mL (Medium dose) and 3.06 × 10^4^ TCID_50_/mL (Low dose). Dose strengths were decided based on dose-dependent mortality outcomes from the literature [28,29] in order to replicate ranavirus infections of varying levels (weak vs. strong). Frogs were then exposed for 6 h via bath immersion and monitored intermittently during this time (volume was assessed beforehand to ensure full coverage of the body (excluding the head). Fluid from the Petri dishes was drained into waste buckets, the frogs removed and placed back into individual housing (controls always handled first).

Crickets were administered to each tank every three days with controls fed first, followed by the low, medium and then high dose treatment groups to minimise the risk of contamination. Daily checks were performed in the same order, to monitor the health of each individual and look for overt clinical signs of ranavirosis: oedema, erythema, petechial haemorrhaging, lethargy, inappetence and ulceration (Table S1). Lids were lifted, with all checks performed and hides removed, if frogs were hidden. Any frogs that displayed possible overt signs of ill health were then inspected more closely.

**Figure 2:**
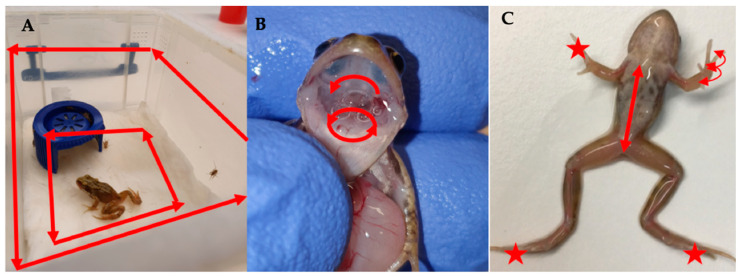
Demonstrating non-lethal sampling protocols for (**A**) Tank swab, (**B**) Buccal swab and (**C**) Body and digit swabs. ★ indicate areas targeted by the digit sampling protocol and arrows indicate the direction of movement of the different swabbing protocols.

Based on evidence in the literature [13,29], we decided to sample on days 2, 4 and 6 post-exposure, in order to capture the pattern of progression of a ranavirus infection, which we predicted would have a dose-dependent pattern of variation. On each of the sampling days, a third of the frogs were removed from each treatment group (7 per treatment), and non-lethal sampling was conducted (Table 1, Figure 2). Tank swabs and stool samples were collected once the frogs had been removed from the tank. Following this non-lethal sampling, *R. temporaria* were euthanised via a schedule 1 method which involved immersion in buffered Tricaine Methanesulfonate (MS222) and a non-schedule 1 confirmation of death via submergence in ethanol. Toe clipping/buccal swabbing was performed post-euthanasia to reduce the stress of the frogs, therefore safeguarding amphibian welfare.

Following storage in ethanol (70%), tissues were sampled for qPCR analysis (Table 1). For the control group, only the liver was analysed, as it is the most commonly sampled tissue for ranavirus detection in the literature due to ranaviruses often targeting this organ during an infection [30]. Tissues were targeted in the ranavirus treatment groups for sampling based on evidence of ranavirus presence in tissues early on in infections [18] or as main sites of ranavirus replication [31].

### 2.5. Experiment 2 (Toads): Detectability of RUK13 and PDE18 in Adult B. bufo

**Figure 3:**
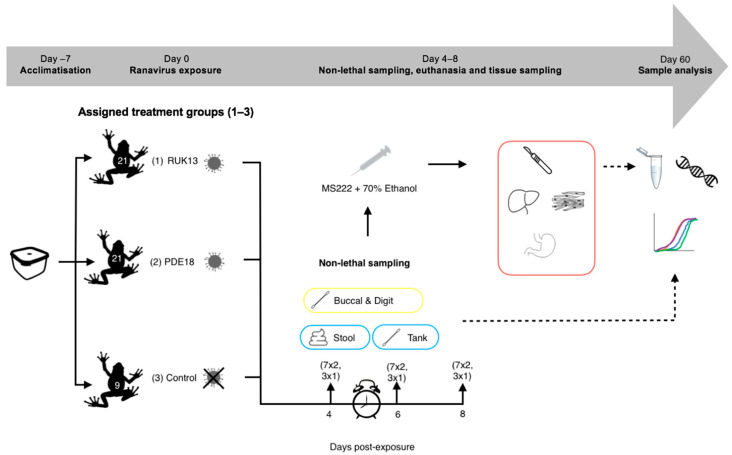
Experimental 2 workflow (experimental design and post-experimental processing).

*Bufo bufo* were transferred to 0.3 L boxes, containing 25 mL of dechlorinated water, according to treatment (Figure 3). 25 mL of RUK13/PDE18 was then added (approx. 6 toads/minute/treatment) to make the final concentration 3.06 × 10^5.5^ TCID_50_/mL. The toads were then exposed via bath immersion for 8 h and monitored intermittently during this time (volume was assessed as in experiment 1). The exposure mixture was then drained from the tanks into waste buckets, the toads removed and placed back into individual housing.

The toads continued to be fed every four days starting with the control group first. The toads were checked daily by eye to monitor their health and to check for clinical signs of ranavirosis (Table S1). A more thorough inspection via handling was conducted when cleaning tanks.

Sampling was conducted on days 4, 6 and 8 post-exposure to ranavirus based on evidence in the literature. When looking at the survivorship of common toad and frog tadpoles when exposed to RUK13, Duffus (2014), found that mortality was seen in common frogs earlier (day 4) then common toads (day 6) [29]. CMTV-like viruses were investigated in smooth newts: mortality and clinical signs of disease were first observed at day 7 post-exposure [13]. Based on these outcomes, it was decided that eight days after exposure would be enough time for both viruses to progress into a strong infection within the toads and that sampling on days 4, 6 and 8 would provide the greatest chance to sample ranavirus both early and later on in infection.

A third of the toads were removed on each sampling day (7 per ranavirus treatment, 3 from control treatment), and conducted non-lethal sampling (Table 2, Figure 2). Prior to euthanasia, tanks were swabbed once the toads were removed, stool collected, and the digits of the toads swabbed. *B. bufo* were then euthanised via a schedule 1 method which involved immersion in buffered Tricaine Methanesulfonate (MS222) and non-schedule one confirmation of death via submergence in ethanol. Buccal swabbing was then performed, followed by sampling the liver, gastro-intestinal tract and leg muscle for qPCR analysis based on performance in experiment 1, commonly sampled tissue in the literature (liver) and areas associated with clinical signs of ranavirosis.

### 2.6. Extraction and Amplification of Viral DNA

DNA was extracted from both experiments using the DNeasy^®^ Blood & Tissue Kit (Qiagen, Hilden, Germany) for all samples (spin-column formats), with the exception of the faecal samples which were extracted using the DNeasy^®^ PowerSoil^®^ DNA extraction Kit (Qiagen). We determined ranavirus presence using a quantitative PCR assay specific to amphibian-associated ranaviruses (AARVs) developed by Leung et al. in 2017 [32]. Whilst currently not part of the accepted methods for ranavirus detection as presented by the OIE, the method has been evaluated in multiple studies; [25,32,33]. The primers used to detect ranavirus targeted a 97 bp region of the viral Major Capsid Protein (MCP) and detection of host DNA to determine viral load of tissues (experiment 1 only), targeted an ultra-conserved single-copy locus in vertebrates (EBF3N).

20 µL reactions were set up containing; 10 µL TaqMan Universal 2× PCR Master Mix, 5.95 µL Nuclease-free water, 1 µL each of 10 µM forward and reverse primers, 0.05 µL of 100 µM probe and 2 µL of DNA template. Samples were placed on 0.1 mL MicroAmp Optical 96-Well Reaction Plates (Thermofisher Scientific, Waltham, MA, USA) alongside 2 no-template controls, 2 negative extractions, 4 ranavirus standards (3 × 10^7^, 10^5^, 10^3^ and 10^1^) and sealed with MicroAmp Optical Adhesive Film (Thermofisher Scientific, Waltham, MA, USA). Plates were run on StepOnePlus Real-Time PCR Systems (Applied Biosystems, Waltham, MA, USA) with the following cycle settings: 50 °C for 2 min, 95 °C for 10 min, and 50 cycles of 95 °C for 15 s and 60 °C for 30 s. Samples were run in duplicate and considered positive if a sigmoidal amplification curve was present in both replicates above the fluorescence threshold, the cycle number at which the fluorescence generated will not be confused with background signal. Any samples in which only one replicate amplified were repeated until both replicates reached a consensus. Viral load, i.e., MCP copies per host cell, was then calculated using the equation outlined in Leung et al. (2017) [32]. We used MCP qPCR values as a measure of infection intensity, these values were normalised for tissues using EBF3N qPCR values to determine viral copies per host cell.

### 2.7. Analysis

In experiment 1 (frogs), we considered an animal infected with ranavirus only if at least two sample types tested positive for ranavirus DNA. Similarly, an animal in which a negative ranavirus DNA result was shared with all other sampling methods or all but one sampling method, was then considered clear of infection at the time of sampling. Sensitivity (true positive rate) and specificity (true negative rate) of each sample type were calculated using the formulas below. We then calculated the false positive (1–specificity) and negative rates (1–sensitivity) for each sample type.

$$\mathrm{Sensitivity}=\frac{\mathrm{number}\;\mathrm{of}\;\mathrm{true}\;\mathrm{positives}}{\mathrm{number}\;\mathrm{of}\;\mathrm{true}\;\mathrm{positives}+\mathrm{number}\;\mathrm{of}\;\mathrm{false}\;\mathrm{negatives}}$$

(1) $$\mathrm{Specificity}=\frac{\mathrm{number}\;\mathrm{of}\;\mathrm{true}\;\mathrm{negatives}}{\mathrm{number}\;\mathrm{of}\;\mathrm{true}\;\mathrm{negatives}+\mathrm{number}\;\mathrm{of}\;\mathrm{false}\;\mathrm{positives}}$$

We compared detectability overall between non-lethal and lethal sampling, between virus exposed toads vs. non-virus exposed toads and compared the number of clinical signs between treatment groups using a fisher’s exact test. Control animals were not included in the statistical analysis detailed below unless specifically stated.

Generalised linear mixed effects models were used to estimate the fixed (day, dose/virus, sample type) and random effects (individual frogs/toads) on ranavirus presence. The models were run in R studio v.1.2.5 using the glmer command in the package “lme4” (optimizer = bobyqa, family = binomial(logit)). To correct for zero-inflated viral load data (as determined using the model diagnostics package “DHARMa”), we used zero-inflated gaussian mixed models to estimate the fixed (dose/virus, sampling day, sample type) and random effects (individual frogs/toads) on the viral load. Models were run in R studio v.1.2.5 using the lme.zig command in the package “NBZIMM”. Post hoc tests were then conducted using the package “emmeans” to estimate marginal means with a Bonferroni correction and determine the best fit model for the data.

## 3. Results

Within the control groups, ranavirus was detected in two frogs and one toad. These results were verified by sequencing, which confirmed the presence of an FV3-like virus. No virus was detected in the negative control extractions. In the frogs, the contamination was external: in a single stool sample and one pre-death swab of stomach and digits, so can be attributed to low levels of cross contamination between containers. In the single toad, the ranavirus was detected more extensively, in all the non-lethal samples and the gastro-intestinal tract. This contamination may be better explained by a spillage of exposure media during the exposure of the experimental animals.

### 3.1. Infection Dynamics in R. temporaria: Screening for Ranavirus Using Tissue

**Figure 4:**
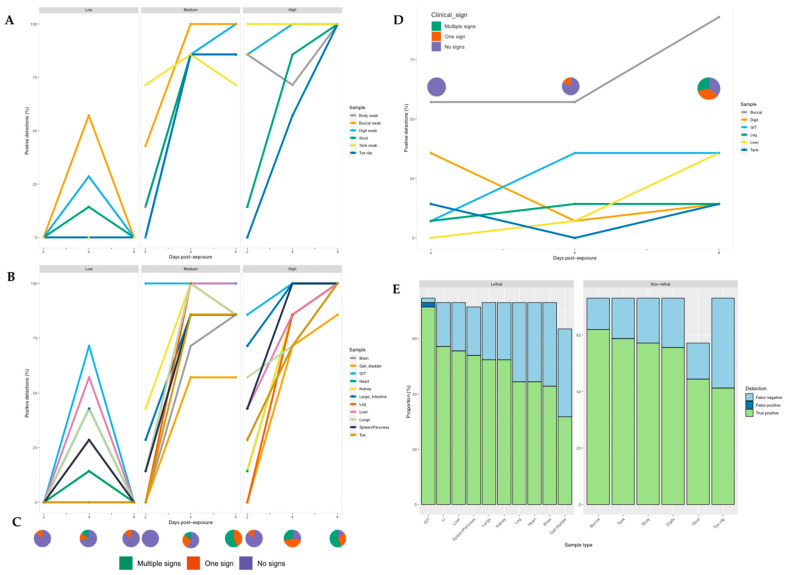
Proportion of samples positive for ranavirus for each individual sampling technique at different time points post-exposure in *R. temporaria* (left and bottom right) and *B. bufo* (top right). (**A**) Non-lethal and (**B**) Lethal sampling post-exposure for each ranavirus dose. (**C**) Clinical signs observed post-exposure across the low, medium and high dose treatment groups. (**D**) Non-lethal and lethal sampling post-exposure in toads. (**E**) Proportion of results that were true positives, false positives and false negatives for non-lethal and lethal ranavirus sampling in frogs.

Detectability: Across the experiment as a whole, we detected ranavirus across all tissues in some frogs. Within the low dose treatment, detection of ranavirus occurred only on the middle sampling day and most prominently in the gastrointestinal tract (GIT) and liver. In frogs exposed to a high virus dose, infection increased across all three days, whereas at intermediate dose it appeared to increase then stall (toe, gallbladder, heart, large intestine) or drop slightly (lung, leg, kidney) by the final sampling day (Figure 4). Detectability was highest in the GIT across all treatment groups and sampling days (Table S2) and exhibited significantly higher viral loads across all doses on the majority of sampling days. Specificity amongst tissues ranged from 94–100% and sensitivity from 50–98% (Table S3).

**Figure 5:**
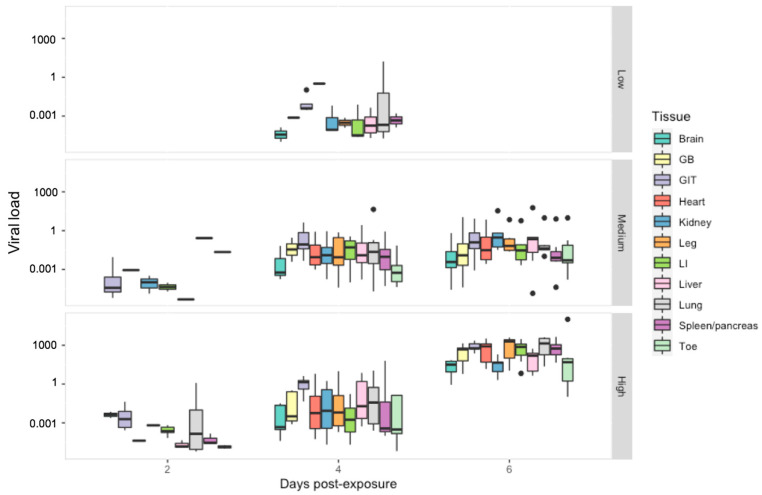
Viral load per host cell for tissue harvested from *R. temporaria* including: brain, gall bladder (GB), gastro-intestinal tract (GIT), heart, kidney, leg, large intestine (LI), liver, lung, spleen/pancreas and toe. Viral load is shown here in log10 scale. Black dots indicate outliers.

Viral Load: When we examined the effect of tissue type on viral load, the gastrointestinal tract had a significantly higher viral load then all other tissue types except the lung (*p* < 0.01) (Figure 5). Viral load overall varied significantly between all ranavirus treatment groups (*p* < 0.01) and sampling days (*p* < 0.03). Within the low dose treatment, tissues with the highest viral load included the lung and heart. In the other treatment groups, the lung maintained consistently high viral loads with notable increases observed amongst the kidney, liver and leg within the intermediate treatment; by the last sampling day viral load amongst most tissues were similar to that of the gastro-intestinal tract in the high dose treatment (Figure S1, Table S6).

### 3.2. Non-Lethal Screening for Ranavirus in R. temporaria

Ranavirus was detected in 75% of frogs exposed to RUK13; one frog died. Ranavirus was detected in 100% and 90% of the detectably infected frogs using lethal and non-lethal sampling, respectively (excluding the control group). Specificity across the non-lethal samples was 100%, whilst sensitivity ranged from 57–85% (Table S3). Of the non-lethal samples, ranavirus was most reliably detected using the buccal swab, followed closely by the tank and body swab; only the gastrointestinal tract demonstrated a higher detection rate (Figure 4). The buccal swab and gastro-intestinal tract assays success rates were not significantly different (Table 3). Similarly, when comparing detection rates between gold standard tissues recommended by the OIE (liver, kidney, spleen, lung, and skin) and non-lethal sampling, swabbing techniques had greater success at screening for ranavirus, while the stool and toe clip samples performed worse (Table S5).

**Figure 6:**
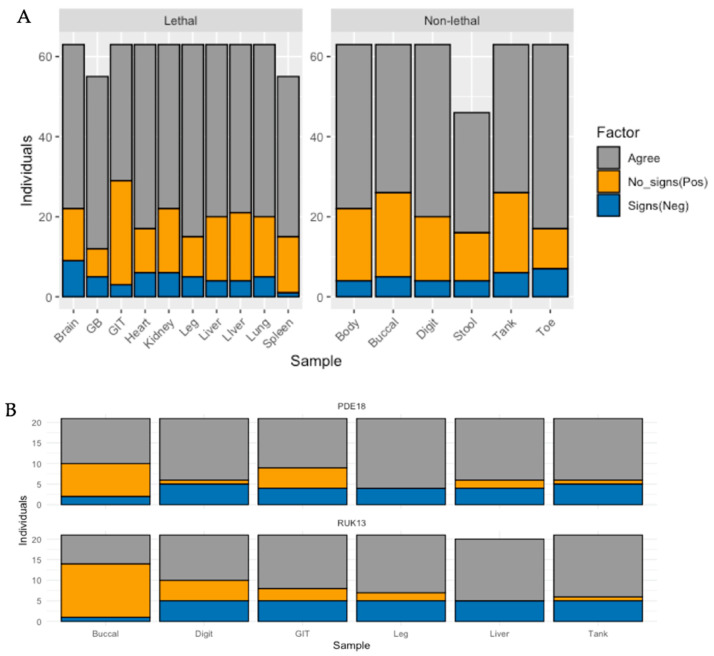
Bar plot illustrating the number of individuals for (**A**) frogs, and (**B**) toads that were; positive for ranavirus with each sample type in the absence of clinical signs (orange), the number of individuals negative for ranavirus by sample type in the presence of clinical signs (blue), and the number of individuals in which sample type agrees with absence/presence of clinical signs (grey).

Whilst detection rates were significantly higher in the medium and high dose groups (*p* < 0.0001), no pronounced difference was observed when comparing the low dose treatment with the control group (*p* = 0.608). Of the frogs exposed to RUK13, 37% developed clinical signs attributed to ranavirus (Table S8). Both lethal and non-lethal sampling detected the virus in a large proportion of cases showing no clinical signs (Figure 6).

Ranavirus quantities in buccal swabs were consistently higher than other non-lethal sample types and either contained similar or higher quantities than the liver, kidney and GIT (Figure S2). Quantities of virus early on in infection were highest within buccal swabs, which, as infection progressed, remained consistently high. In comparison, virus in tissues began, on average, at lower levels, and increased to quantities similar or slightly higher than in the swab by the last sampling day (Table S11).

### 3.3. Ranavirus Shedding in R. temporaria

**Figure 7:**
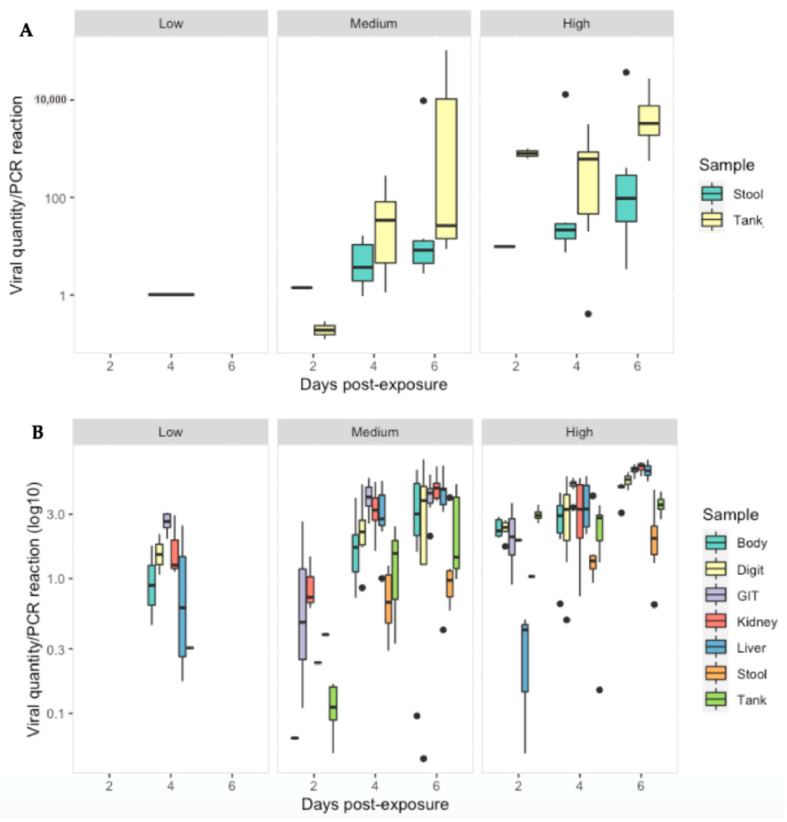
Viral quantity per PCR reaction for: (**A**) stool and tank swabs and (**B**) Body, digit and tank swabs, GIT, kidney and liver tissue obtained from *R. temporaria* from all treatment groups at 2, 4 and 6 days-post exposure to ranavirus. Black dots indicate outliers.

Ranavirus was detected in 28/40 (70%) of stool samples and 37/63 (59%) of tank swabs. Similar to tissues, viral quantity increased significantly with increasing dose (*p* < 0.01). The low dose treatment group had significantly lower viral quantities then the other treatment groups across all sampling days (*p* < 0.05). Significant interactions, indicating further increases on later days at higher doses were detected in some combinations (Table S7). Ranavirus quantity (per PCR reaction) was substantially less in the stool and tank swabs compared to the gastro-intestinal tract (*p* < 0.0001), liver and kidney (*p* < 0.003) as infection progressed in the medium/high dose treatments (Figure 7).

### 3.4. Screening for RUK13 and PDE18 in Toads

**Figure 8:**
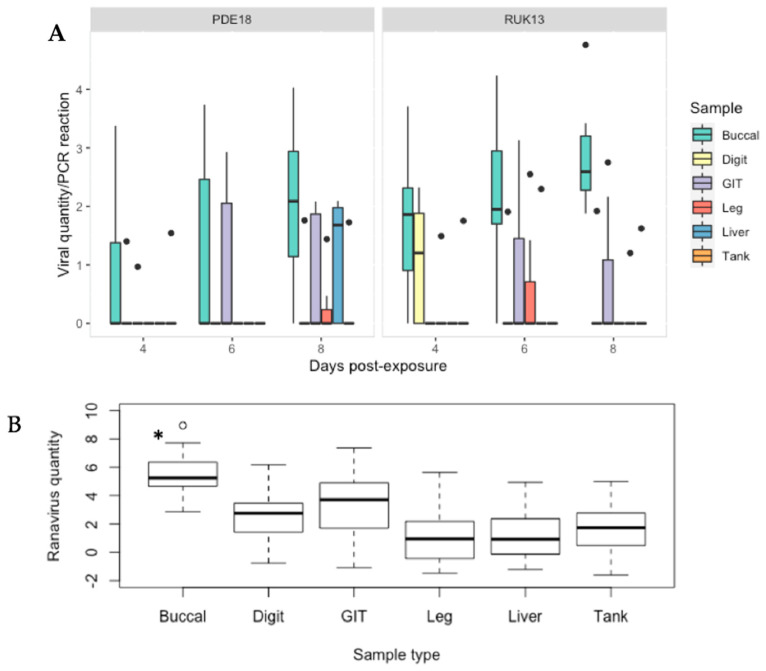
(**A**) Viral quantity per PCR reaction for buccal, digit and tank swabs, GIT, leg and liver tissue obtained from *B. bufo* from both virus treatment groups at 4, 6 and 8 days-post exposure. (**B**) Predicted probabilities of ranavirus quantities per 2 μL PCR reaction generated from the zero inflated gaussian mixed model for sample type as a single factor. Black dots indicate outliers. * *p* < 0.0001.

The buccal swabbing assay significantly outperformed all other assays when detecting RUK13 in toads (Table 4). Detection via buccal swabbing remained high in toads exposed to PDE18 though was not significantly higher than other sampling methods (Figure S3). The gastro-intestinal tract remained the tissue with the highest rate of positives in both virus treatment groups (Table S4). A large portion of toads did not produce stool samples throughout the duration of the experiment (83%), and of those that did, no ranavirus was detected. Because of this small sample size, we decided to exclude stool as a sample type from any further analysis. The buccal swabs were also found to contain significantly higher viral quantities than all other sample types (*p* < 0.0001); the gastro-intestinal tract had substantially higher quantities of virus than the other tissues screened (*p* < 0.03; Figure 8). Of the 36% of toads in which ranavirus was present in one or more tissues, 87% of these toads also tested positive by one or more non-lethal sampling methods. In the other 13%, ranavirus was only detected in the gastro-intestinal tract. Ranavirus was detected using non-lethal sampling in 50% of toads (Figure 4D); the buccal swab was the lone detector in 67% of these toads. Similarly to experiment 1, the buccal swab had the highest detection rate in the absence of clinical signs of the non-lethal sampling assays, and in this case of all sampling assays (Figure 6; Figure S4).

There was no significant difference in detectability between the two ranavirus strains (*p* > 0.05), or day of infection (Table S9). Similarly, viral quantity overall, as determined by qPCR, did not differ significantly between viral treatment groups (*p* = 0.12). The buccal swab retained the highest amount of virus across all sampling days within FV3; in the CMTV samples, the relative quantity increased with time post exposure, reaching significantly higher levels of virus by day 6. RUK13 and PDE18 were detected in 2/21 (10%) of tank swabs from each group. Only 29% of exposed toads developed overt clinical signs of ranavirosis (Table S10). No significant difference was found between the presence of clinical signs and the rate of virus detection in any assay.

## 4. Discussion

In this study we set out to identify a non-lethal sampling technique which would detect ranavirus in all types of infections at any stage, without the need for euthanasia. We tested these methods using two ranaviruses from European lineages, RUK13 (FV3-like) and PDE18 (CMTV-like), and two UK-native amphibian species (*R. temporaria* and *B. bufo*) with differing susceptibilities to ranavirus.

Of the non-lethal methods we evaluated, screening for ranavirus using the buccal swab had the highest success for both RUK13 and PDE18. This assay had a comparable performance to lethal tissue sampling approaches in frogs, and outperformed them in toads. The case for non-lethal sampling is reinforced by the demonstration that buccal swab surveys can detect infections which have not produced clinical signs. It could therefore be used in surveys of wild populations to detect virus circulation within frog populations which; (1) appear free of disease and, consequently, would not justify lethal sampling, and (2) be more widely suitable for toad populations which appear to be more resilient to the disease, but which could harbor the virus. Whilst the stool and toe-clip assays were unreliable screening tools for ranavirus particularly in toads, several other non-lethal sampling swabbing techniques were comparable or outperformed the liver-assay, currently one of the tissues recommended as gold standard by the OIE [6].

We found the performance of sampling techniques depended on the intensity of infection, as has previously been demonstrated [9]. The ability of buccal swabbing to detect ranavirus through disease progression, after infections initiated by both medium and high dose of virus, suggest it could be used for screening early on in clinical infections, when disease may not have yet manifested. However, low-level infections, equivalent to sub-clinical infections in the wild, have a more moderate chance of being detected, with successful sampling depending on the stage of the infection. It is currently unclear whether these low-level infections were actually cleared, or remained at levels undetectable using qPCR; though clinical signs, specifically reddening of the skin, observed towards the end of the experiment might indicate that the infection was still present, or the result of post-infection innate immunity [34]. On the other hand, these signs were also seen in the control group and in other animal experiments run under the same rearing conditions at the Zoological Society of London, which we have come to suspect might be associated with damp toweling used in these experiments.

Our investigation of lethal assays using multiple tissues demonstrates that focusing on any single tissue would be misleading, especially when screening for both clinical and subclinical infections. With the exception of the tissues involved in the route of transmission into the host (GI tract and lung), no clear progression of the disease through the organs was determined in frogs. In the OIE manual for infection with ranavirus, it is stated that the best tissues for analysis are the liver, kidney, spleen, lung and skin [6]. However, the previous knowledge on which these recommendations were based was obtained from studies including animals at severe disease endpoints [4,19,20]. We have demonstrated that both ranavirus presence and quantity can vary depending on the stage/intensity of infection and tissue, as hypothesised by Price and colleagues [12]. The buccal swab assay, in contrast, was consistent from early stages of infection, particularly in frogs exposed to medium or high concentrations of ranavirus. Based on this, we recommend that the buccal swab become standard practice for ranavirus screening, and that the gastro-intestinal tract be targeted for ranavirus when tissue samples are necessary.

Both buccal swabbing and toe clipping was performed post-mortem in this study to avoid unnecessary stress and safeguard amphibian welfare. Recently, buccal swabbing has been used in genetic studies of wild amphibian populations, in replace of toe clipping and has been proven to be reliable for microsatellite sequencing capturing good quality DNA [35]. Whilst we believe that, when undertaken by experienced experimenters, buccal swabbing of live amphibians would be expected to perform similarly when viral sampling, this still needs to be validated, both in a laboratory setting and in the wild.

The presence of virus in the buccal swab and gastrointestinal tract, along with higher viral loads, suggest the route of infection into frogs and toads was through the ingestion of virus during the bath exposure. This suggestion is in line with Saucedo et al. (2019) [13], who identified the first targets for CMTV ranavirus in smooth newts, as the oral cavity and respiratory mucosa, as well as other studies [18,36]. Direct ingestion of virus has been shown to be the most virulent route of transmission, causing rapid onset of mortality in tadpoles [28,37]. Amphibians in the wild can ingest virus-infected carcasses leading to the rapid spread of ranavirus in wild populations. However, modelling transmission dynamics of ranaviruses present in common frog populations has demonstrated that ranaviruses in the UK may persist in the short-term solely through adult-to-adult transmission [38]. The route of exposure has shown to result in difference disease outcomes and therefore the transmission route may impact the involvement between ranaviruses and the oral cavity and, therefore, may affect the screening ability of the buccal swab [27]. Further work will need to be conducted to assess the effect of transmission route on buccal swab sampling.

The low level of viral shedding detected in toads may help explain why only a small number of toads are reported with ranavirus in the UK [5] and why ranaviruses were unlikely to be sustained in toad populations without the presence of common frogs [39]. Among common frogs, ranavirus prevalence has been found to decrease with the presence of toads, thought to be the result of less effective transmission and a dilution effect [40]. This may be the case for FV3 in the UK, but in Spain, mass mortalities of common toads due to the CMTV viral lineage have been observed [41]. Only two cases of CMTV have been reported in the UK currently so little is known about transmission and disease dynamics [25]. Our findings showed no significant difference between ranaviruses when examining shedding, signs of disease or detectability overall in toads. We also demonstrated that the buccal swab assay was able to detect both FV3 and CMTV viruses with equal ability, outperforming the lethal assays, and so will prove useful as a tool in determining the true prevalence of CMTV viruses in the UK.

## 5. Conclusions

We examined multiple tissue types in this study to determine true prevalence of ranavirus infection and therefore more accurately predict the sensitivity and specificity of the non-lethal sampling strategies. We used the presence/absence of clinical signs to highlight the severity of infection and tested all non-lethal sampling techniques across different stages and intensities of infection; as well as in species exposed to multiple ranaviruses, at different life stages, with differing susceptibilities to ranavirus. It is our opinion that the culmination of all these analyses provide a thorough and robust evaluation of the ability of these sampling techniques to detect ranavirus.

We found buccal swabbing to be successful at detecting ranavirus, not only in the absence of clinical signs, but also throughout different stages and intensities of infections. Whilst weaker infections still remain challenging to screen, buccal swabbing proved capable of screening for ranavirus(es) in amphibian species of varying susceptibility. Buccal swabbing performed better than currently recommended tissues for sampling demonstrating its suitability as a sampling method for the screening of ranaviruses. Further work will be needed to validate this non-lethal sampling technique, with a focus on the effect of buccal swabbing live amphibians in the UK, as well as targeting other non-UK amphibian species and the different life stages. With further validation, we hope that this screening technique will become standard practice for ranavirus detection, both in the field and within the trade, and help gain a better grasp on ranavirus prevalence both within the UK, and globally.

## Figures and Tables

**Table 1:** Sampling techniques undertaken on *R. temporaria* throughout the experiment. (H) = high dose, (M) = medium dose, (L) = Low dose, (C) = Control.

	Category			Individuals Sampled			Procedure	Storage Prior to Extraction
**Sample type**	Lethal	Destructive	Invasive	Day 2	Day 4	Day 6		
Pre-death swab (control only)			x	7	7	7	**Pre-euthanasia** x 5 over stomach and each digit	4 °C
Buccal swab			x	(H) 7 (M) 7 (L) 7 (C) 6	(H) 7 (M) 7 (L) 7 (C) 6	(H) 7 (M) 7 (L) 7 (C) 6	**Post-euthanasia** x 5 inside the mouth and around the tongue	4 °C
Digit swab			x	(H) 7 (M) 7 (L) 7	(H) 7 (M) 7 (L) 7	(H) 7 (M) 7 (L) 7	**Pre-euthanasia** x 5 over each digit	4 °C
Body swab			x	(H) 7 (M) 7 (L) 7	(H) 7 (M) 7 (L) 7	(H) 7 (M) 7 (L) 7	**Pre-euthanasia** x 5 over the stomach	4 °C
Environmental (tank) swab				(H) 7 (M) 7 (L) 7	(H) 7 (M) 7 (L) 7	(H) 7 (M) 7 (L) 7	**Pre-euthanasia** x 5 over the walls and floor of the housing	4 °C
Stool				(H) 7 (M) 7 (L) 7	(H) 7 (M) 7 (L) 7	(H) 7 (M) 7 (L) 7	**Pre-euthanasia** Collected using forceps	−20 °C
Toe clip		x	x	(H) 7 (M) 7 (L) 7	(H) 7 (M) 7 (L) 7	(H) 7 (M) 7 (L) 7	**Post-euthanasia** Remove fourth right toe of the right foot	70% Ethanol
Tissue (e.g., liver)	x			(H) 7 (M) 7 (L) 7	(H) 7 (M) 7 (L) 7	(H) 7 (M) 7 (L) 7	**Post-euthanasia** Liver, Gastro-intestinal tract, Leg & (13) Tongue	70% Ethanol

**Table 2:** Sampling techniques undertaken on *B. bufo* throughout the experiment. (R) = RUK13, (P) = PDE18, (C) = Control.

	Category			Individuals Sampled			Procedure	Storage Prior to Extraction
**Sample type**	Lethal	Destructive	Invasive	Day 4	Day 6	Day 8		
Buccal swab			x	(R) 7 (P) 7 (C) 3	(R) 7 (P) 7 (C) 3	(R) 7 (P) 7 (C) 3	**Post-euthanasia** x 5 inside the mouth and around the tongue	4 °C
Digit swab			x	(R) 7 (P) 7 (C) 3	(R) 7 (P) 7 (C) 3	(R) 7 (P) 7 (C) 3	**Pre-euthanasia** x 5 over each digit	4 °C
Tank swab				(R) 7 (P) 7 (C) 3	((R) 7 (P) 7 (C) 3	(R) 7 (P) 7 (C) 3	**Pre-euthanasia** x 5 over the walls and floor of the housing	4 °C
Stool				(R) 7 (P) 7 (C) 3	(R) 7 (P) 7 (C) 3	(R) 7 (P) 7 (C) 3	**Pre-euthanasia** Collected using forceps	−20 °C
Tissue (e.g., liver)	x			(R) 7 (P) 7 (C) 3	(R) 7 (P) 7 (C) 3	(R) 7 (P) 7 (C) 3	**Post-euthanasia** Liver, Gastro-intestinal tract, Leg & (13) Tongue	70% Ethanol

**Table 3:** Sample types ranked by detection rate (%) with significant differences of pairwise comparisons between sample types illustrated.

Assay (Ranked by Rate)	Acronym	Detection Rate %	Significant Differences															
			GIT	BS	TS	BdS	LI	DS	LV	S/P	LU	KD	LG	HT	ST	BR	TC	GB
Gastro-intestinal tract	GIT	71		NS	NS	NS	NS	NS	NS	*	**	**	***	***	*	***	***	***
Buccal swab	BS	62			NS	NS	NS	NS	NS	NS	NS	NS	NS	NS	NS	*	**	***
Tank swab	TS	59				NS	NS	NS	NS	NS	NS	NS	NS	NS	NS	NS	NS	**
Body swab	BdS	57					NS	NS	NS	NS	NS	NS	NS	NS	NS	NS	NS	*
Large intestine	LI	57						NS	NS	NS	NS	NS	NS	NS	NS	NS	NS	*
Digit swab	DS	56							NS	NS	NS	NS	NS	NS	NS	NS	NS	*
Liver	TV	56								NS	NS	NS	NS	NS	NS	NS	NS	*
Spleen/pancreas	S/P	54									NS	NS	NS	NS	NS	NS	NS	NS
Lungs	LU	52										NS	NS	NS	NS	NS	NS	NS
Kidney	KD	52											NS	NS	NS	NS	NS	NS
Leg	LG	44												NS	NS	NS	NS	NS
Heart	HT	44													NS	NS	NS	NS
Stool	ST	44														NS	NS	NS
Brain	BR	43															NS	NS
Toe clip	TC	41																NS
Gall bladder	GB	32																

* ** *** = *p* < 0.05, 0.01, 0.001; NS = Not Significant.

**Table 4:** Sample types ranked by detection rate (%) with significant differences of pairwise comparisons between sample types illustrated overall and by virus type.

Assay (Ranked by Rate)	Detection Rate %			Significant Differences														
	RUK13	PDE18	Total	GIT	DS	LV	LG	TS	GIT	DS	LV	LG	TS	GIT	DS	LV	LG	TS
Buccal swab (BS)	86	57	71	***	***	***	***	***	**	*	**	**	**	NS	NS	NS	NS	NS
Gastro-intestinal tract (GIT)	28	33	26		NS	NS	NS	NS		NS	NS	NS	NS		NS	NS	NS	NS
Digit swab (DS)	24	1	19			NS	NS	NS			NS	NS	NS			NS	NS	NS
Liver (LV)	1	19	14	Total			NS	NS	RUK13			NS	NS	PDE18			NS	NS
Leg (LG)	14	1	12					NS					NS					NS
Tank swab (TS)	1	1	10															

* ** *** = *p* < 0.05, 0.01, 0.001; NS = Not Significant.

## Data Availability

Any computer code used to generate results reported in the manuscript as well as raw data that support the findings of this study are available on request from the corresponding author, without undue reservation.

## Supplementary Materials

The following supporting information can be downloaded at: https://www.mdpi.com/article/10.3390/v14122635/s1, Figure S1: Predicted probabilities of viral load in *R. temporaria* generated from the zero inflated gaussian mixed model; Figure S2: Predicted probabilities of ranavirus quantity/PCR reaction in *R. temporaria* generated from the zero inflated gaussian mixed model; Figure S3: Predicted probabilities of outcome generated from generalised linear mixed effects model of toads; Figure S4: Clinical signs observed in *B. bufo* for both ranavirus treatment groups; Table S1: Clinical signs of ranavirosis [42]; Table S2: Detection of RUK13 in *R. temporaria*; Table S3: Sample type performance in *R. temporaria*; Table S4: Detection of RUK13 and PDE18 in *B. bufo*; Table S5: Generalised Linear Mixed effects model output for detectability in *R. temporaria*; Table S6: Zero-inflated Gaussian Mixed effects model output for viral load in *R. temporaria*; Table S7: Zero-inflated Gaussian Mixed effects model outputs for viral quantity during shedding in *R. temporaria*; Table S8: Clinical signs observed in frogs for the duration of the experiment; Table S9: Mixed effects model p-values for fixed effects and their interactions in toads; Table S10: Clinical signs observed in toads for the duration of the experiment; Table S11: Zero-inflated Gaussian Mixed effects model outputs for viral quantity in *R. temporaria* (Quantity ~ Sample*Day*Dose + (1|Frog)) *p*-values for fixed effects and their interactions in buccal, body and tank swabs, along with toe clips, stool, liver, GIT and kidney tissue.

## Abbreviations

PPE (Personal protective equipment), TCID_50_ (Median Tissue Culture Infectious Dose).
