# Supplementary material for: Non-Lethal Detection of Frog Virus 3-Like (RUK13) and Common Midwife Toad Virus-Like (PDE18) Ranaviruses in Two UK-Native Amphibian Species
Source: Viruses. 2022 Nov 25;14(12):2635. doi: 10.3390/v14122635 (PMC9786228; doi:10.3390/v14122635)
Supplement: Supplementary file 1 [file viruses-14-02635-s001.zip › viruses-2022475-supplementary.pdf]

Supplementary information

Table S1. Clinical signs of ranavirosis. A list of clinical signs of ranavirosis that were watched for throughout this study as well as humane end-point checks considered in the thesis by Brookes, 2022 (Brookes, 2022).

Sign	Description
Ranavirosis	
Oedema	The physical appearance of an enlarged area, with tightening of the skin increasing with an increase severity of oedema. Caused by excess fluid acclimating under the skin. Area affected is specific to the anatomy: -dermis around the mandible (offered referred to as lips), floor of oral cavity, dermis around the eye, and broader areas such as the abdomen, drink patch, upper front limbs and upper hindlimbs, digits and cloaca. Blood vesicles will be constricted by the increasing fluid under the skin and will be contributed, appearing bright red. The area affected by oedema may appear both rigid and floppy dependent on where the oedema is. The presence of the fluid can be confirmed by a physical exam or by histopathological assessment.
Tremors	When the amphibian's muscles are in spasm, overtly clear in the hind limbs and sometimes eyes and muscles around the face. Can last for a few seconds and each recovery and new event recorded separately.
Swelling	Inflammation of a localised area, appearing as a small bump in a part of an individual's external appearance. Will disappear with the reversal of the inflammatory response and can be manipulated on an individual's exterior.
Erythema	Can appear on any part of the body<br>The skin appears with generalised reddened skin caused by increased blood flow and vessel congestion in the skin or mucous membranes.
Haemorrhage	Can appear on any part of the body<br>**Petechiae:** bleeding into the skin/tissue and gives the appearance of tiny red dots, that can be seen on their own or in clusters.<br>**Ecchymosis:** Bleeding occurs as a result of blood vessels. Gives the appearance of a large bruise.
Lethargic/laboured movements	**Generalised:** Slowed, infrequent and/or abnormal movements and other behaviours performed abnormally, slowly and less frequently than unaffected animals. Reduced use of space, with possibly a reduction in the normal physical range of movement.<br>**Laboured hopping/jumping**<br>**Laboured walking**
Emaciation	When an animal loses weight (and in the absence of being weighed, when an individual shrink's in appearance with a decrease in a scored body condition but with no change in length of growth stage).<br>Includes the defined use of 'anorexia', which infers a physiological element
Lack of appetite	Summarised, as an individual showing no attempt to seek out food when offered and/or by the weight/quantity of leftover food (those sizable to ingest).
Humane end-point checks:	
Apathy	**General:** A lack of enthusiasm or interest.<br>**Delay or loss of hop reflex**<br>**Decrease in activity**<br>**Loss or decrease in antipredator response**<br>**Loss of aggressive behaviour**<br>**A loss of a startle response**<br>**Reduced responsiveness to being handled**<br>**Obtunded**
Poor righting reflex	When inverted: i) fails to place all four limbs back on the ground and support its own body weight; ii) laboured righting attempts, often with hind limbs extended and uncoordinated limb movements; iii) marked decrease in reaction to inversion/increase in reaction time when inverted, effects increasing with each invert. Tested 3 times.
Uncoordinated	Loss of balance and/or coordination during locomotion (walk, hop, climb) and unusual and slow eye movements, lacking in focus. Can also affect feeding behaviours, with difficulty focusing on food, may result in less successful strike for post-metamorphic or land-based amphibians. Behaviour is usually unchanged when overtly stimulated.

Table S2. Detection of RUK13 in *R.temporaria* at day 2, 4, and 6 post-exposure using quantitative PCR (qPCR) for samples obtained pre and post-euthanasia. Positive (+), negative (-), sample not done (ND).

	No. of days post-infection											
	**Day 2**				**Day 4**				**Day 6**			
	Control	Low	Medium	High	Control	Low	Medium	High	Control	Low	Medium	High
**Swab**												
Pre-death	7(-)				1(+), 6(-)				7(-)			
Buccal	6(-), 1(ND)	7(-)	3(+), 4(-)	6(+), 1(-)	6(-), 1(ND)	4(+), 3(-)	7(+)	7(+)	6(-), 1(ND)	7(-)	7(+)	7(+)
Digits		7(-)	7(-)	6(+), 1(-)		2(+), 5(-)	6(+), 1(-)	7(+)		7(-)	7(+)	7(+)
Body		7(-)	1(+), 6(-)	6(+), 1(-)		2(+), 5(-)	6(+), 1(-)	5(+), 2(-)		7(-)	7(+)	7(+)
Environment	7(-)	7(-)	5(+), 2(-)	7(+)	7(-)	7(-)	6(+), 1(-)	7(+)	7(-)	7(-)	5(+), 2(-)	7(+)
**Stool**	4(-), 3(ND)	2(-), 5(ND)	2(+), 5(ND)	(1+), 1(-), 5(ND)	1(+), 5(-), 1(ND)	1(+), 5(-), 1(ND)	6(+), 1(-)	6(+), 1(ND)	7(-)	7(-)	6(+), 1(-)	7(+)
**Toe clip**		7(-)	7(-)	7(-)		7(-)	6(+), 1(-)	4(+), 3(-)		7(-)	6(+), 1(-)	7(+)
**Liver**	7(-)	7(-)	1(+), 6(-)	3(+), 4(-)	7(-)	4(+), 3(-)	7(+)	6(+), 1(-)	7(-)	7(-)	7(+)	7(+)
**Kidney**		6(-), 1(ND)	1(+), 6(-)	1(+), 6(-)		3(+), 4(-)	7(+)	6(+), 1(-)		7(-)	6(+), 1(-)	7(+)
**Brain**	1(-), 6(ND)	7(-)	7(-)	2(+), 5(-)	1(-), 6(ND)	2(+), 5(-)	5(+), 2(-)	5(+), 2(-)	1(-), 6(ND)	7(-)	6(+), 1(-)	7(+)
**GIT**	1(-), 6(ND)	7(-)	7(+)	6(+), 1(-)	1(-), 6(ND)	5(+), 2(-)	7(+)	7(+)	1(-), 6(ND)	7(-)	7(+)	7(+)
**Gall bladder**		6(-), 1(ND)	6(-), 1(ND)	7(-)		1(+), 6(-)	4(+), 2(-), 1(ND)	5(+), 2(-)		7(-)	4(+), 3(ND)	6(+), 1(ND)
**Heart**	1(-), 6(ND)	7(-)	1(+), 6(-)	1(+), 6(-)	1(-), 6(ND)	1(+), 6(-)	6(+), 1(-)	6(+), 1(-)	1(-), 6(ND)	7(-)	6(+), 1(-)	7(+)
**Lungs**	1(-), 6(ND)	7(-)	1(+), 6(-)	4(+), 3(-)	1(-), 6(ND)	3(+), 4(-)	7(+)	5(+), 2(-)	1(-), 6(ND)	7(-)	6(+), 1(-)	7(+)
**Leg**	1(-), 6(ND)	7(-)	7(-)	7(-)	1(-), 6(ND)	2(+), 5(-)	7(+)	6(+), 1(-)	1(-), 6(ND)	7(-)	6(+), 1(-)	7(+)
**Spleen/Pancreas**	1(-), 6(ND)	7(-)	1(+), 1(-), 5(ND)	3(+), 4(-)	1(-), 6(ND)	2(+), 5(-)	7(+)	7(+)	1(-), 6(ND)	7(-)	7(+)	7(+)
**Large Intestine**	1(-), 6(ND)	7(-)	2(+), 5(-)	5(+), 2(-)	1(-), 6(ND)	3(+), 4(-)	6(+), 1(-)	7(+)	1(-), 6(ND)	7(-)	6(+), 1(-)	7(+)

Table S3. Sample type performance in *R. temporaria*. The performance of each sample type across all three sampling days and treatments in frogs using: test sensitivity and specificity and the rate of false negatives (β) and false positives (**α**).

	% detectability	Sensitivity (%)	Specificity (%)	α (false +ve) %	β (false -ve) %
**Swab**					
Buccal	62	85	100	0	15
Digits	56	76	100	0	24
Body	57	78	100	0	22
Tank	59	80	100	0	20
**Stool**	44	78	100	0	22
**Toe clip**	41	57	100	0	43

	% detectability	Sensitivity (%)	Specificity (%)	α (false +ve) %	β (false -ve) %
Liver	56	76	100	0	24
Brain	43	59	100	0	39
Gastro-intestinal tract	71	98	94	6	2
Gall bladder	32	50	100	0	50
Kidney	52	72	100	0	25
Heart	44	61	100	0	37
Lungs	52	72	100	0	25
Leg	44	61	100	0	37
Spleen/Pancreas	54	76	100	0	21
Large Intestine	57	78	100	0	19

Table S4. Detection of RUK13 and PDE18 in *B.bufo* at day 4, 6, and 8 post-exposure using quantitative PCR (qPCR) for samples obtained pre and post-euthanasia. Positive (+), negative (-), sample not done (ND).

	**Day 4**			**Day 6**			**Day 8**		
	Control	PDE18	RUK13	Control	PDE18	RUK13	Control	PDE18	RUK13
**Swab**									
Tank	1(+), 2(-)	1(+), 6(-)	1(+), 6(-)	3(-)	7(-)	7(N)	3(-)	1(+), 6(-)	7(-)
Digit	1(+), 2(-)	1(+), 6(-)	3(+), 4(-)	3(-)	7(-)	1(+), 6(-)	3(-)	1(+), 6(-)	1(+), 6(-)
Buccal	1(+), 2(-)	2(+), 5(-)	5(+), 2(-)	3(-)	2(+), 5(-)	5(+), 2(-)	3(-)	5(+), 2(-)	7(+)
**Stool**	3(ND)	7(ND)	7(ND)	1(-), 2(ND)	7(ND)	5(-), 2(ND)	1(-), 2(ND)	1(-), 6(ND)	1(-), 6(ND)
**Liver**	3(-)	7(-)	7(-)	3(-)	7(-)	1(+), 6(-)	3(-)	4(+), 3(-)	1(+), 6(-)
**GIT**	1(+), 2(-)	7(-)	7(-)	3(-)	3(+), 4(-)	2(+), 5(-)	3(-)	2(+), 5(-)	2(+), 5(-)
**Leg**	3(-)	7(-)	1(+), 6(-)	3(-)	7(-)	2(+), 5(-)	3(-)	2(+), 5(-)	7(-)

Table S5. Generalised Linear Mixed effects model output for *R. temporaria* (Outcome ~ Sample + Day + Dose + (1|Frog)) p-values for fixed effects and their interactions with detectability. An effect is deemed significant if p-value is <0.05.

Fixed effect	-	-	p value
Dose	Low	Medium	<0.0001
		High	<0.0001
	Medium	High	0.6046
Day	2	4	<0.0001
		6	<0.0001
	4	6	1
Sample	Buccal	Brain	0.0221
		Gall bladder	0.0005
		Toe clip	0.0066
	Digits	Gall bladder	0.0454
	Body	Gall bladder	0.0159
	Brain	GIT	<.0001
	Gastro-intestinal tract	Gall bladder	<.0001
		Heart	<.0001
		Kidney	0.007
		Leg	<.0001
		Lung	0.007
		Spleen/pancreas	0.0262
		Stool	0.0108
		Toe	<.0001
	Gall bladder	Large Intestine	0.0159
		Liver	0.0454
		Tank	0.0053

Table S6. Zero-inflated Gaussian Mixed effects model output for viral load in *R. temporaria* (Load ~ Tissue*Dose*Day + (1|Frog)) p-values for fixed effects and their interactions with viral load of tissues. An effect is deemed significant if p-value is <0.05.

Fixed effect	-	-	p value
Day	2	4	<0.0001
		6	<0.0001
	4	6	0.0227
Dose	Low	Medium	<0.0001
		High	<0.0001
	Medium	High	0.0006
Organ	Brain	GIT	**<.0001**
		Lung	0.0001
	Gall bladder	GIT	**<.0001**
		Lung	0.0003
	GIT	Heart	0.0008
		Kidney	0.0001
		Leg	0.0001
		LI	0.0021
		Liver	<0.0001
		Spleen/pancreas	0.002
		Toe	<0.0001
	Heart	Toe	0.0354
	LI	Toe	0.0052
	Lung	Toe	<0.0001
	Spleen/pancreas	Toe	0.0092
Day*Dose	Day 4 - Low	Day 4 - Medium	0.0228
		Day 4 - High	0.0468
	Day 6 - Low	Day 6 - Medium	<0.0001
		Day 6 - High	<0.0001
	Day 2 - Medium	Day 4 - Medium	0.0015
		Day 6 - Medium	0.0003
	Day 6 - Medium	Day 6 - High	<0.0001
	Day 2 - High	Day 4 - High	0.0371
		Day 6 - High	<0.0001
	Day 4 - High	Day 6 - High	<0.0001
Dose*Organ	Medium - Brain	Medium - GIT	<0.0001
		Medium - Kidney	0.019
		Medium - LI	0.0205
		Medium - Lung	<0.0001
		Medium - Spleen/pancreas	0.0206
	Medium - GB	Medium - GIT	0.0001
		Medium - Lung	0.0028
	Medium - GIT	Medium - Heart	0.008
		Medium - Kidney	0.0096
		Medium - Liver	<0.0001
		Medium - Toe	<0.0001
	Medium - Kidney	Medium - Toe	0.0385
	Medium - LI	Medium - Lung	0.0071
	Medium - Lung	Medium - Toe	<0.0001
	Medium - Spleen/pancreas	Medium - Toe	0.0399
	High - Brain	High - GIT	<0.0001
		High - Lung	0.0027
	High - GB	High - GIT	<0.0001
	High - GIT	High - Heart	<0.0001
		High - Kidney	<0.0001
		High - Leg	<0.0001
		High - LI	<0.0001
		High - Liver	<0.0001
		High - Lung	<0.0001
		High - Spleen/pancreas	<0.0001
		High - Toe	<0.0001
	High - LI	High - Lung	<0.0001
	High - Liver	High - Lung	0.015
	High - Lung	High - Toe	<0.0001
	High - Spleen/pancreas	High - Toe	0.0002

Fixed effect	-	-	p value
Organ*Day	Day 2 - GB	Day 6 - GB	0.0005
	Day 2 - GIT	Day 4 - GIT	<0.0001
		Day 6 - GIT	0.002
	Day 2 - Heart	Day 4 - Heart	0.0039
		Day 6 - Heart	0.0001
	Day 2 - Kidney	Day 4 - Kidney	0.0222
		Day 6 - Kidney	0.0003
	Day 2 - Leg	Day 4 - Leg	0.0032
		Day 6 - Leg	<0.0001
	Day 2 - LI	Day 6 - LI	0.0084
	Day 2 - Liver	Day 4 - Liver	<0.0001
		Day 6 - Liver	<0.0001
	Day 2 - Lung	Day 6 - Lung	0.0321
	Day 2 - Spleen/pancreas	Day 6 - Spleen/pancreas	0.0031
	Day 2 - Toe	Day 6 - Toe	0.0055
	Day 4 - Brain	Day 4 - GIT	<0.0001
		Day 4 - Heart	0.0003
		Day 4 - Kidney	0.002
		Day 4 - Leg	0.0043
		Day 4 - Liver	<0.0001
		Day 4 - Lung	<0.0001
	Day 4 - GB	Day 4 - GIT	<0.0001
		Day 4 - Lung	0.0091
	Day 4 - GIT	Day 4 - Heart	<0.0001
		Day 4 - Kidney	<0.0001
		Day 4 - Leg	<0.0001
		Day 4 - LI	<0.0001
		Day 4 - Liver	<0.0001
		Day 4 - Spleen/pancreas	<0.0001
		Day 4 - Toe	<0.0001
	Day 4 - Heart	Day 4 - Toe	0.0016
	Day 4 - Kidney	Day 4 - Toe	0.0118
	Day 4 - Leg	Day 4 - Toe	0.0147
	Day 4 - Liver	Day 4 - Toe	0.0001
	Day 4 - Lung	Day 4 - Toe	<0.0001

Fixed effect	-	-	p value
Organ l Day l Dose	Day 4 - Low<br>Brain	Day 4 - Low<br>GIT	<0.0001
	Day 4 - Low<br>GB	Day 4 - Low<br>GIT	<0.0001
		Day 4 - Low<br>Lung	0.0076
	Day 4 - Low<br>GIT	Day 4 - Low<br>Kidney	0.0003
		Day 4 - Low<br>Leg	0.0001
		Day 4 - Low<br>LI	<0.0001
		Day 4 - Low<br>Liver	0.0001
		Day 4 - Low<br>Speen/pancreas	0.0001
		Day 4 - Low<br>Toe	0.0128
	Day 2 - Medium<br>Kidney	Day 2 - Medium<br>Lung	0.0007
	Day 2 - Medium<br>LI	Day 2 - Medium<br>Liver	0.008
	Day 2 - Medium<br>Liver	Day 2 - Medium<br>Lung	<0.0001
		Day 2 - Medium<br>Spleen/pancreas	0.0005
	Day 4 - Medium<br>Brain	Day 4 - Medium<br>GIT	<0.0001
		Day 4 - Medium<br>Heart	0.0275
		Day 4 - Medium<br>Kidney	0.0003
		Day 4 - Medium<br>Leg	0.0002
		Day 4 - Medium<br>LI	0.0002
		Day 4 - Medium<br>Liver	<0.0001
		Day 4 - Medium<br>Lung	<0.0001
		Day 4 - Medium<br>Spleen/pancreas	0.018
	Day 4 - Medium<br>Gall bladder	Day 4 - Medium<br>GIT	0.0012
		Day 4 - Medium<br>Toe	0.0026
	Day 4 - Medium<br>GIT	Day 4 - Medium<br>Heart	0.0002
		Day 4 - Medium<br>Kidney	0.0058
		Day 4 - Medium<br>Leg	0.0119
		Day 4 - Medium<br>LI	0.0425
		Day 4 - Medium<br>Spleen/pancreas	0.0001
		Day 4 - Medium<br>Toe	<0.0001
	Day 4 - Medium<br>Heart	Day 4 - Medium<br>Toe	0.0002
	Day 4 - Medium<br>Kidney	Day 4 - Medium<br>Toe	<0.0001
	Day 4 - Medium<br>Leg	Day 4 - Medium<br>Toe	<0.0001
	Day 4 - Medium<br>LI	Day 4 - Medium<br>Toe	<0.0001
	Day 4 - Medium<br>Liver	Day 4 - Medium<br>Toe	<0.0001

Fixed effect	-	-	p value
Organ∣Day∣Dose	Day 4 - Medium Lung	Day 4 - Medium Toe	<0.0001
	Day 4 - Medium Spleen/pancreas	Day 4 - Medium Toe	0.0001
	Day 6 - Medium Brain	Day 6 - Medium GIT	<0.0001
		Day 6 - Medium Heart	0.0158
		Day 6 - Medium Kidney	<0.0001
		Day 6 - Medium Leg	<0.0001
		Day 6 - Medium Liver	<0.0001
		Day 6 - Medium Lung	0.0008
		Day 6 - Medium Spleen/pancreas	0.0418
	Day 6 - Medium Gall bladder	Day 6 - Medium GIT	<0.0001
		Day 6 - Medium Kidney	<0.0001
		Day 6 - Medium Leg	0.001
		Day 6 - Medium Liver	<0.0001
		Day 6 - Medium Lung	0.0125
	Day 6 - Medium GIT	Day 6 - Medium Heart	0.0427
		Day 6 - Medium LI	0.0019
		Day 6 - Medium Spleen/pancreas	0.0084
		Day 6 - Medium Toe	<0.0001
	Day 6 - Mediun Kidney	Day 6 - Medium LI	0.0137
	Day 6 - Mediun Leg	Day 6 - Medium Toe	0.0404
	Day 2 - High Brain	Day 2 - High Liver	0.0007
	Day 2 - High Gall bladder	Day 2 - High GIT	0.0045
	Day 2 - High GIT	Day 2 - High Heart	<0.0001
		Day 2 - High Kidney	0.0342
		Day 2 - High Leg	0.0045
		Day 2 - High LI	0.0451
		Day 2 - High Liver	<0.0001
		Day 2 - High Spleen/pancreas	<0.0001
		Day 2 - High Toe	<0.0001
	Day 2 - High LI	Day 2 - High Liver	0.0001
	Day 2 - High Liver	Day 2 - High Lung	0.0003
	Day 4 - High Brain	Day 4 - High GB	0.0414
		Day 4 - High GIT	<0.0001
		Day 4 - High Heart	0.0052

Fixed effect	-	-	p value
Organ|Day|Dose		Day 4 - High Kidney	0.0057
		Day 4 - High Leg	0.0001
		Day 4 - High Liver	<0.0001
		Day 4 - High Lung	0.0002
		Day 4 - High Spleen/pancreas	0.0011
	Day 4 - High Gall bladder	Day 4 - High GIT	<0.0001
	Day 4 - High GIT	Day 4 - High Heart	<0.0001
		Day 4 - High Kidney	<0.0001
		Day 4 - High Leg	<0.0001
		Day 4 - High LI	<0.0001
		Day 4 - High Liver	<0.0001
		Day 4 - High Lung	<0.0001
		Day 4 - High Spleen/pancreas	<0.0001
		Day 4 - High Toe	<0.0001
	Day 4 - High LI	Day 4 - High Liver	0.0032
	Day 4 - High Liver	Day 4 - High Toe	<0.0001
	Day 4 - High Lung	Day 4 - High Toe	0.0025
	Day 4 - High Spleen/pancreas	Day 4 - High Toe	0.017
	Day 6 - High Brain	Day 6 - High GIT	<0.0001
		Day 6 - High Heart	0.0006
		Day 6 - High Leg	<0.0001
		Day 6 - High LI	0.0001
		Day 6 - High Lung	<0.0001
		Day 6 - High Spleen/pancreas	<0.0001
	Day 6 - High Gall bladder	Day 6 - High Toe	0.0018
	Day 6 - High GIT	Day 6 - High Kidney	<0.0001
		Day 6 - High Liver	0.0028
		Day 6 - High Toe	<0.0001
	Day 6 - High Heart	Day 6 - High Kidney	0.0041
		Day 6 - High Toe	<0.0001
	Day 6 - High Kidney	Day 6 - High Leg	0.0001
		Day 6 - High LI	0.0011
		Day 6 - High Lung	<0.0001
		Day 6 - High Spleen/pancreas	0.0001

Fixed effect	-	-	p value
Organ|Day|Dose	Day 6 - High<br>Leg	Day 6 - High<br>Liver	0.0436
		Day 6 - High<br>Toe	<0.0001
	Day 6 - High<br>Liver	Day 6 - High<br>Lung	0.0074
	Day 6 - High<br>Lung	Day 6 - High<br>Toe	<0.0001
	Day 6 - High<br>Spleen/pancreas	Day 6 - High<br>Toe	<0.0001

Table S7. Zero-inflated Gaussian Mixed effects model outputs for viral quantity during shedding *in R. temporaria* (Quantity ~ Sample*Day*Dose + (1|Frog)) p-values for fixed effects and their interactions with viral shedding in stool and tank swabs. An effect is deemed significant if p-value is <0.05.

Fixed effect	-	-	p value
Day	2	4	<0.0001
		6	<0.0001
Dose	Low	Medium	<0.0001
		High	<0.0001
	Medium	High	<0.0001
Day*Dose	Day 2 - Low	Day 4 - Low	<0.0001
		Day 2 - Medium	0.0002
		Day 2 - High	<0.0001
	Day 4 - Low	Day 4 - Medium	0.0005
		Day 4 - High	<0.0001
		Day 6 - Low	<0.0001
	Day 6 - Low	Day 6 - Medium	<0.0001
		Day 6 - High	<0.0001
	Day 2 - Medium	Day 4 - Medium	<0.0001
		Day 6 - Medium	<0.0001
		Day 2 - High	0.0036
	Day 6 - Medium	Day 6 - High	0.0457
	Day 2 - High	Day 6 - High	<0.0001
	Day 4 - High	Day 6 - High	0.0295
Sample | Day	Day 4 - GIT	Day 4 - Body	<0.0001
		Day 4 - Digit	<0.0001
		Day 4 - Kidney	0.0009
		Day 4 - Liver	0.0001
		Day 4 - Stool	<0.0001
		Day 4 - Tank	<0.0001
	Day 4 - Body	Day 4 - Tank	0.0073
	Day 4 - Digit	Day 4 - Stool	0.0085
		Day 4 - Tank	0.0003
	Day 4 - Kidney	Day 4 - Stool	<0.0001
		Day 4 - Tank	<0.0001
	Day 4 - Liver	Day 4 - Stool	0.0001
		Day 4 - Tank	<0.0001
	Day 6 - GIT	Day 6 - Stool	0.0209
	Day 6 - Kidney	Day 6 - Stool	0.0107
	Day 6 - Liver	Day 6 - Stool	0.025
	Day 2 - Stool	Day 4 - Stool	0.0009
Dose*Sample	Medium - Body	Medium - GIT	<0.0001
		Medium - Kidney	0.0099
	Medium - Digit	Medium - GIT	<0.0001
		Medium - Kidney	0.0002
		Medium - Liver	0.0361
	Medium - GIT	Medium - Liver	0.0486
		Medium - Stool	<0.0001
		Medium - Tank	<0.0001
	Medium - Kidney	Medium - Stool	<0.0001
		Medium - Tank	<0.0001
	Medium - Liver	Medium - Stool	0.0013
	High - Body	High - GIT	0.0003
		High - Stool	<0.0001
	High - Digit	High - Stool	<0.0001
	High - GIT	High - Kidney	0.01
		High - Liver	0.0002
		High - Stool	<0.0001
		High - Tank	<0.0001
	High - Kidney	High - Stool	0.0002
	High - Liver	High - Stool	<0.0001
	High - Stool	High - Tank	<0.0001
	Low - Stool	Medium - Stool	<0.0001
		High - Stool	<0.0001
	Low - Tank	Medium - Tank	<0.0001
		High - Tank	<0.0001
	Medium - Tank	High - Tank	<0.0001

Fixed effect	-	-	p value
Sample ǀ Dose ǀ Day	Day 2 - Medium Digit	Day 2 - Medium GIT	0.0006
		Day 2 - Medium Kidney	0.008
		Day 2 - Medium Tank	0.0256
	Day 2 - High Body	Day 2 - High Liver	0.0002
		Day 2 - High Stool	0.0331
	Day 2 - High Digit	Day 2 - High Liver	0.0002
		Day 2 - High Stool	0.0313
	Day 2 - High GIT	Day 2 - High Liver	0.0008
	Day 2 - High Kidney	Day 2 - High Tank	0.004
	Day 2 - High Liver	Day 2 - High Tank	<0.0001
	Day 2 - High Stool	Day 2 - High Tank	0.0012
	Day 4 - Low Body	Day 4 - Low Tank	0.0197
	Day 4 - Low Digit	Day 4 - Low Tank	0.0065
	Day 4 - Low GIT	Day 4 - Low Liver	0.025
		Day 4 - Low Stool	0.0045
		Day 4 - Low Tank	<0.0001
	Day 4 - Low Kidney	Day 4 - Low Tank	0.0021
	Day 4 - Low Liver	Day 4 - Low Tank	0.007
	Day 4 - Medium Body	Day 4 - Medium GIT	0.0109
		Day 4 - Medium Liver	0.0304
	Day 4 - Medium GIT	Day 4 - Medium Digit	0.0034
		Day 4 - Medium Stool	<0.0001
		Day 4 - Medium Tank	<0.0001
	Day 4 - Medium Kidney	Day 4 - Medium Stool	<0.0001
		Day 4 - Medium Tank	0.0002
	Day 4 - Medium Liver	Day 4 - Medium Stool	<0.0001
		Day 4 - Medium Tank	0.0006
	Day 4 - High GIT	Day 4 - High Body	0.0003
		Day 4 - High Digit	0.0064
		Day 4 - High Kidney	0.0054
		Day 4 - High Stool	<0.0001
		Day 4 - High Tank	<0.0001
	Day 4 - High Liver	Day 4 - High Stool	0.023
	Day 6 - Medium Body	Day 6 - Medium Stool	<0.0001

Fixed effect	-	-	p value
	Day 6 - Medium<br>Digit	Day 6 - Medium<br>Stool	<0.0001
	Day 6 - Medium<br>GIT	Day 6 - Medium<br>Stool	<0.0001
	Day 6 - Medium<br>Kidney	Day 6 - Medium<br>Stool	<0.0001
		Day 6 - Medium<br>Tank	0.0002
	Day 6 - Medium<br>Liver	Day 6 - Medium<br>Stool	<0.0001
		Day 6 - Medium<br>Tank	0.001
	Day 6 - High<br>Body	Day 6 - High<br>GIT	0.0058
		Day 6 - High<br>Kidney	0.0004
		Day 6 - High<br>Liver	0.0064
		Day 6 - High<br>Stool	<0.0001
	Day 6 - High<br>Digit	Day 6 - High<br>Stool	<0.0001
		Day 6 - High<br>Tank	0.005
	Day 6 - High<br>GIT	Day 6 - High<br>Stool	<0.0001
		Day 6 - High<br>Tank	<0.0001
	Day 6 - High<br>Kidney	Day 6 - High<br>Stool	<0.0001
		Day 6 - High<br>Tank	<0.0001
	Day 6 - High<br>Liver	Day 6 - High<br>Stool	<0.0001
		Day 6 - High<br>Tank	<0.0001
	Day 2 - Low<br>Stool	Day 2 - High<br>Stool	0.0171
	Day 4 - Low<br>Stool	Day 4 - High<br>Stool	0.0109
	Day 6 - Low<br>Stool	Day 6 - Medium<br>Stool	0.0095
		Day 6 - High<br>Stool	<0.0001
	Day 2 - Low<br>Tank	Day 2 - Medium<br>Tank	0.0095
		Day 2- High<br>Tank	<0.0001
	Day 2 - Medium<br>Tank	Day 2- High<br>Tank	<0.0001
	Day 4 - Low<br>Tank	Day 4 - Medium<br>Tank	0.0003
		Day 4 - High<br>Tank	<0.0001
	Day 6 - Low<br>Tank	Day 6 - Medium<br>Tank	0.0001
		Day 6 - High<br>Tank	<0.0001

Table S8. Clinical signs observed in frogs for the duration of the experiment.

Treatment	Frog	Sign	Days post-exposure
	A4/1/1		
	A4/1/2		
	A4/1/3		
	A4/1/4		
	A4/1/5		
	A4/1/6		
	B4/1		
	A4/2/1		
	A4/2/2		
	A4/2/3		
Control	A4/2/4	Red skin on stomach	4
	A4/2/5		
	A4/2/6		
	B4/2		
	A4/3/1		
	A4/3/2		
	A4/3/3		
	A4/3/4		
	A4/3/5		
	A4/3/6		
	B4/3		
	B3/1/1		
	B3/1/2		
	B3/1/3	Red Cloaca	2
	B3/1/4		
	B3/1/5		
	B3/1/6		
	B3/1/7	Red thighs	2
	B3/2/1	Loss of appetite	4
	B3/2/2		
Low	B3/2/3	Prolapsed cloaca<br>Redness on right arm	4
	B3/2/4		
	B3/2/5		
	B3/2/6		
	B3/2/7		
	B3/3/1		
	B3/3/2		
	B3/3/3		
	B3/3/4		
	B3/3/5	Redness of arms and digits	6
	B3/3/6		
	B3/3/7		
	B2/1/1		
	B2/1/2		
	B2/1/3		
	B2/1/4		
	B2/1/5		
	B2/1/6		
	B2/1/7		
	B2/2/1		
	B2/2/2		
Medium	B2/2/3	Redness near right eye	4
	B2/2/4	Sitting lethargically	3
		Prolapsed cloaca<br>H. top of left leg	4
	B2/2/5	Swelling at Cloaca	3
	B2/2/6		
	B2/2/7		
	B2/3/1	Swollen stomach<br>Redness on feet<br>Leg erythema	6
	B2/3/2	Lip erythema	6

Treatment	Frog	Sign	Days post-exposure
Medium	B2/3/3	Leg erythema<br>Swollen stomach<br>Redness on upper right arm<br>Lip erythema	6
	B2/3/4	Lip erythema	6
	B2/3/5	Leg erythema	6
	B2/3/6	Red tongue<br>Redness on lower abdomen and feet	6
	B2/3/7		
High	B1/1/1		
	B1/1/2		
	B1/1/3		
	B1/1/4	Loss of appetite	2
	B1/1/5		
	B1/1/6		
	B1/1/7		
	B1/2/1	Loss of appetite	4
	B1/2/2		
	B1/2/3		
	B1/2/4	Red digits	4
	B1/2/5	Loss of appetite	3
		Bleeding cloaca	4
	B1/2/6	Loss of appetite	4
		Red tongue	4
	B1/2/7	Red tongue	4
	B1/3/1	Loss of appetite	4
		Red digits<br>Red mouth	6
	B1/3/2	Loss of appetite	4
		Bloated stomach	5
		MORT<br>Single red digit<br>Red cloaca	6
	B1/3/3	Loss of appetite	4
		Leg erythmia<br>Lip eryhtmia	6
	B1/3/4		
	B1/3/5	Loss of appetite	4
		Leg erythmia<br>Lip erythmia	6
	B1/3/6	Loss of appetite	4
	B1/3/7	Red left eye	6

Table S9. Mixed effects model p-values for fixed effects and their interactions in toads. An effect is deemed significant if p-value is <0.05.

Fixed effect	-	-	p value
Virus	CMTV	FV3	0.1236
Day	Day 4	Day 6	1
		Day 8	0.3607
	Day 6	Day 8	1
Sample	Buccal	Digit	<0.0001
		GIT	<0.0001
		Leg	<0.0001
		Liver	<0.0001
		Tank	<0.0001
	GIT	Leg	0.0013
		Liver	0.0042
		Tank	0.0336
Virus*Sample	CMTV - Buccal	CMTV - Digit	0.0001
		CMTV - GIT	0.023
		CMTV - Leg	0.017
		CMTV Liver	<0.0001
		CMTV - Tank	0.0001
	FV3 - Buccal	FV3 - Digit	0.0007
		FV3 - GIT	0.0024
		FV3 - Leg	<0.0001
		FV3 - Liver	<0.0001
		FV3 - Tank	<0.0001
	CMTV - GIT	CMTV - Leg	0.0151
Sample∣Day∣Virus	CMTV - Day 6 Buccal	CMTV - Day 6 Digit	0.0068
		CMTV - Day 6 Leg	0.0068
		CMTV - Day 6 Liver	0.0068
		CMTV - Day 6 Tank	0.0068
	CMTV - Day 8 Buccal	CMTV - Day 8 Digit	0.0055
		CMTV - Day 8 Leg	<0.0001
		CMTV - Day 8 Tank	0.0043
	CMTV - Day 8 GIT	CMTV - Day 8 Leg	0.0028
	CMTV - Day 8 Leg	CMTV - Day 8 Liver	0.002
	FV3 - Day 4 Buccal	FV3 - Day 4 GIT	0.0047
		FV3 - Day 4 Leg	0.0047
		FV3 - Day 4 Liver	0.0047
		FV3 - Day 4 Tank	0.0047
	FV3 - Day 6 Buccal	FV3 - Day 6 Liver	0.008
		FV3 - Day 6 Tank	0.0022
	FV3 - Day 6 GIT	FV3 - Day 6 Tank	0.0165
	FV3 - Day 8 Buccal	FV3 - Day 8 Digit	0.0022
		FV3 - Day 8 Leg	0.0001
		FV3 - Day 8 Liver	<0.0001
		FV3 - Day 8 Tank	0.0002
	FV3 - Day 8 GIT	FV3 - Day 8 Leg	0.019
		FV3 - Day 8 Liver	0.001

Table S10. Clinical signs observed in toads for the duration of the experiment.

Treatment	Toad	Gender	Sign	Day post-exposure
	C1/1	Male		
	C1/2	Male		
	C1/3	Male		
	C2/1	Female	Red left digit	6
	C2/2	Female		
Control	C2/3	Female		
	C3/1	Male	v. slight redness on foot (right)	6
	C3/2	Female		
	C3/3	Male	Red spots on roof of mouth	8
	R1/1	Male		
	R1/2	Female		
	R1/3	Male		
	R1/4	Male		
	R1/5	Male		
	R1/6	Female		
	R1/7	Male		
	R2/1	Female	Spots on roof/back of mouth<br>Red spotty tongue<br>Red eye (right)	6
	R2/2	Male	Red spot on tongue/ reddened at base	6
	R2/3	Female		
	R2/4	Female		
RUK13	R2/5	Male		
	R2/6	Female		
	R2/7	Female		
	R3/1	Male	Gaping	7
			Red tongue	8
	R3/2	Male	x1 cricket (inappetance)	3
			Red webbing (right foot)<br>red tongue<br>red leg (right and bit on left)<br>haemorrhage	8
	R3/3	Female	Spotty tongue	8
	R3/4	Female		
	R3/5	Female		
	R3/6	Female		
	R3/7	Female	lip erythema	6
	P1/1	Female		
	P1/2	Male		
	P1/3	Male		
	P1/4	Male		
	P1/5	Male		
	P1/6	Female		
	P1/7	Female		
	P2/1	Female		
	P2/2	Female		
	P2/3	Female		
	P2/4	Female		
PDE18	P2/5	Male	Red tongue	6
	P2/6	Female		
	P2/7	Female		
	P3/1	Female	Spotty tongue	
	P3/2	Male	x1 (inappetance)	
	P3/3	Female	x2 (inappetance)	
	P3/4	Female		
	P3/5	Male	Lethargic	2
			Red spotted tongue	8
	P3/6	Male	Red abdomen and upper legs	8
	P3/7	Female		

Table S11. Zero-inflated Gaussian Mixed effects model outputs for viral quantity in *R. temporaria* (Quantity ~ Sample*Day*Dose + (1|Frog)) p-values for fixed effects and their interactions in buccal, body and tank swabs, along with toe clips, stool, liver, GIT and kidney tissue. An effect is deemed significant if p-value is <0.05. Only the interactions of Sample*Day*Dose are shown below.

Fixed effect interactions		P value
Day 4 – Low Body	Day 4 – Low Buccal	0.0293
	Day 4 – Low Toe clip	0.0465
	Day 4 – Low Tank	0.0465
Day 4 – Low Buccal	Day 4 – Low Liver	0.0110
	Day 4 – Low Stool	0.0009
	Day 4 – Low Toe clip	<0.0001
	Day 4 – Low Tank	<0.0001
Day 4 – Low GIT	Day 4 – Low Stool	0.0123
	Day 4 – Low Toe clip	0.0002
	Day 4 – Low Tank	0.0002
Day 4 – Low Kidney	Day 4 – Low Toe clip	0.0053
	Day 4 – Low Tank	0.0053
Day 4 – Low Liver	Day 4 – Low Toe clip	0.0180
	Day 4 – Low Tank	0.0180
Day 2 – Medium Buccal	Day 2 – Medium Digit	0.0151
	Day 2 – Medium Toe clip	0.0151
Day 2 – Medium Digit	Day 2 – Medium GIT	0.0021
	Day 2 – Medium Kidney	0.0289
Day 2 – Medium GIT	Day 2 – Medium Toe clip	0.0021
Day 2 – Medium Kidney	Day 2 – Medium Toe clip	0.0289
Day 4 – Medium Body	Day 4 – Medium GIT	0.0001
	Day 4 – Medium Kidney	0.0353
Day 4 – Medium Buccal	Day 4 – Medium Stool	0.0003
	Day 4 – Medium Toe clip	0.0284
	Day 4 – Medium Tank	0.0056
Day 4 – Medium Digit	Day 4 – Medium GIT	0.0121

Fixed effect interactions		P value
Day 4 – Medium GIT	Day 4 – Medium Stool	<0.0001
	Day 4 – Medium Toe clip	<0.0001
	Day 4 – Medium Tank	<0.0001
Day 4 – Medium Kidney	Day 4 – Medium Stool	<0.0001
	Day 4 – Medium Toe clip	0.0044
	Day 4 – Medium Tank	0.0007
Day 4 – Medium Liver	Day 4 – Medium Stool	0.0001
	Day 4 – Medium Toe clip	0.0128
	Day 4 – Medium Tank	0.0023
Day 6 – Medium Body	Day 6 – Medium Stool	0.0001
Day 6 – Medium Buccal	Day 6 – Medium Stool	<0.0001
	Day 6 – Medium Toe clip	0.0247
	Day 6 – Medium Tank	0.0026
Day 6 – Medium Digit	Day 6 – Medium Stool	0.0002
Day 6 – Medium GIT	Day 6 – Medium Stool	<0.0001
	Day 6 – Medium Toe clip	0.0168
	Day 6 – Medium Tank	00017
Day 6 – Medium Kidney	Day 6 – Medium Stool	<0.0001
	Day 6 – Medium Toe clip	0.0093
	Day 6 – Medium Tank	0.0010
Day 6 – Medium Liver	Day 6 – Medium Stool	<0.0001
	Day 6 – Medium Toe clip	0.0341
	Day 6 – Medium Tank	0.0037
Day 2 – High Body	Day 2 – High Liver	0.0010
	Day 2 – High Toe clip	0.0036

Fixed effect interactions		P value
Day 2 – High Buccal	Day 2 – High Kidney	0.0025
	Day 2 – High Liver	<0.0001
	Day 2 – High Stool	0.0005
	Day 2 – High Toe clip	<0.0001
Day 2 – High Digit	Day 2 – High Liver	0.0013
	Day 2 – High Toe clip	0.0046
Day 2 – High GIT	Day 2 – High Liver	0.0050
	Day 2 – High Toe clip	0.0078
Day 2 – High Kidney	Day 2 – High Tank	0.0178
Day 2 – High Liver	Day 2 – High Tank	<0.0001
Day 2 – High Stool	Day 2 – High Tank	0.0043
Day 2 – High Toe clip	Day 2 – High Tank	<0.0001
Day 4 – High Body	Day 4 – High Buccal	0.0028
	Day 4 – High GIT	0.0010
Day 4 – High Buccal	Day 4 – High Digit	0.0366
	Day 4 – High Kidney	0.0239
	Day 4 – High Stool	<0.0001
	Day 4 – High Tank	0.0001
Day 4 – High Digit	Day 4 – High GIT	0.0200
Day 4 – High GIT	Day 4 – High Kidney	0.0151
	Day 4 – High Stool	<0.0001
	Day 4 – High Tank	<0.0001

Fixed effect interactions		P value
Day 6 – High Body	Day 6 – High GIT	0.0182
	Day 6 – High Kidney	0.0016
	Day 6 – High Liver	0.0200
	Day 6 – High Stool	0.0001
	Day 6 – High Toe clip	0.0103
Day 6 – High Buccal	Day 6 – High Stool	<0.0001
	Day 6 – High Tank	0.0001
Day 6 – High Digit	Day 6 – High Stool	<0.0001
	Day 6 – High Tank	0.0158
Day 6 – High GIT	Day 6 – High Stool	<0.0001
	Day 6 – High Tank	<0.0001
Day 6 – High Kidney	Day 6 – High Stool	<0.0001
	Day 6 – High Tank	<0.0001
Day 6 – High Liver	Day 6 – High Stool	<0.0001
	Day 6 – High Tank	<0.0001
Day 6 – High Stool	Day 6 – High Tank	<0.0001
Day 6 – High Toe clip	Day 6 – High Tank	<0.0001

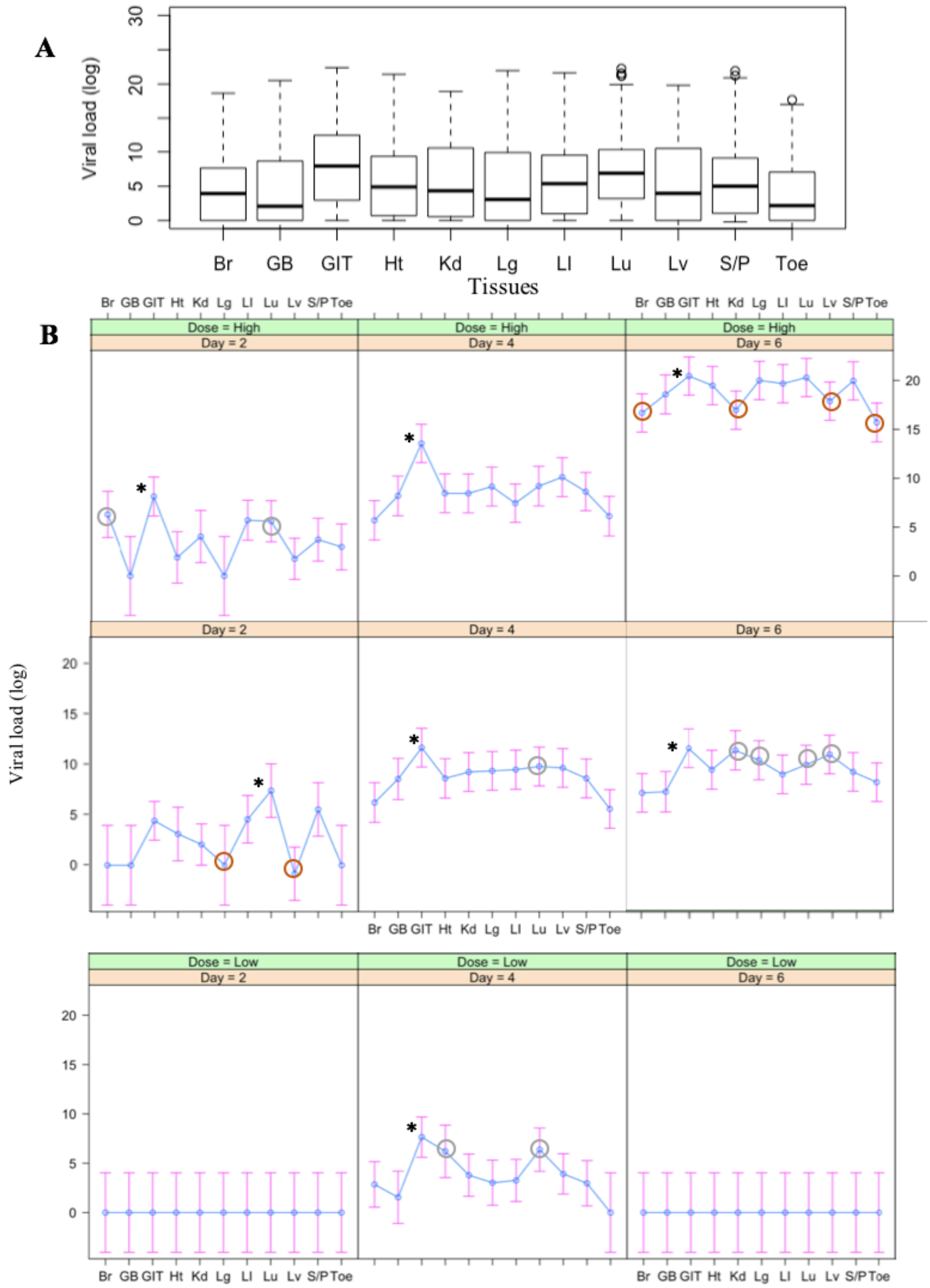

Figure S1. Predicted probabilities of viral load in *R.temporaria* generated from the zero inflated gaussian mixed model for A) tissue as a single factor and, B) tissue*day*dose as interactions. Br=brain, GB=gall bladder, GIT=gastro-intestinal tract, Ht=heart, Kd=kidney, Lg=leg muscle, LI=large intestine, Lu=lung, Lv=liver, S/P=spleen/pancreas, Toe=toe clip. * indicates a tissue with significantly higher viral loads than either tissue ringed in red, or all tissues not ringed in grey.

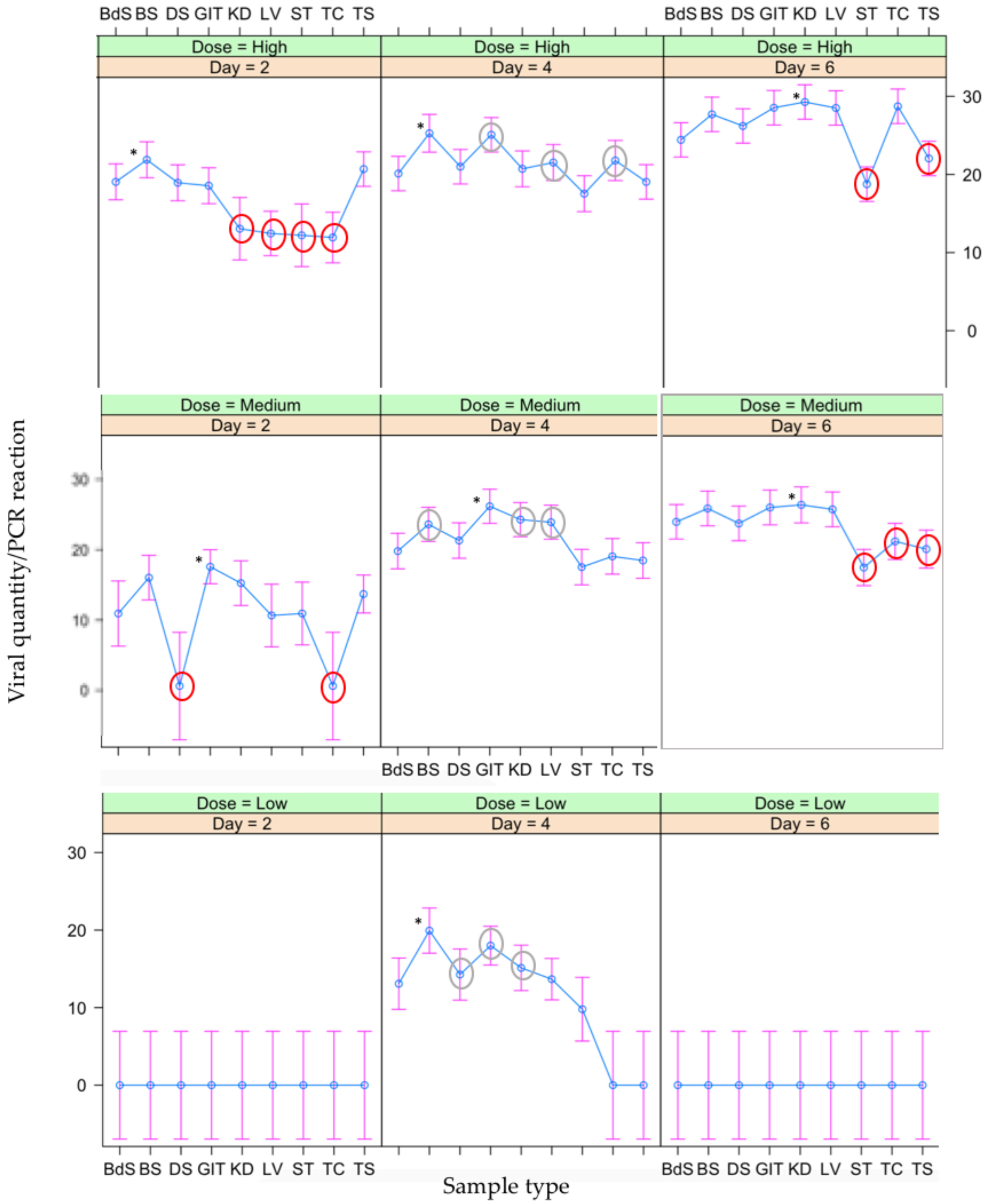

Figure S2. Predicted probabilities of ranavirus quantity/PCR reaction in *R.temporaria* generated from the zero inflated gaussian mixed model for sample*day*dose as interactions. BdS=body swab, BS=buccal swab, DS=digit swab, GIT=gastro-intestinal tract, Kd=kidney, Lv=liver, ST=stool, TC=toe clip, TS=tank swab. * indicates a sample with significantly higher quantities or virus than either tissue ringed in red, or all tissues not ringed in grey.

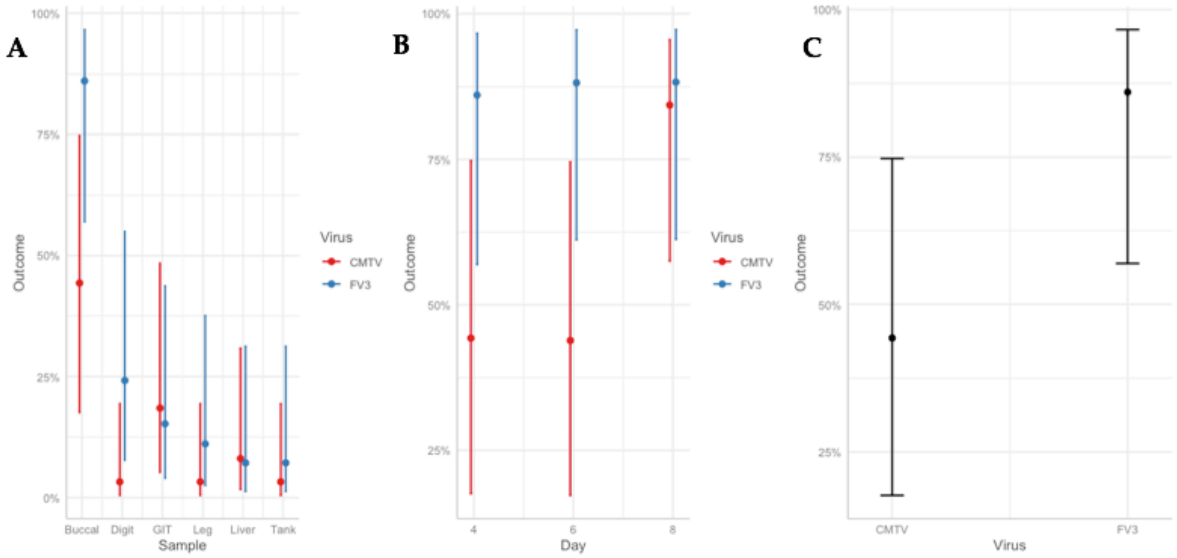

Figure S3. Predicted probabilities of outcome generated from generalised linear mixed effects model of toads for a) sample type with virus, b) virus with day and c) virus only.

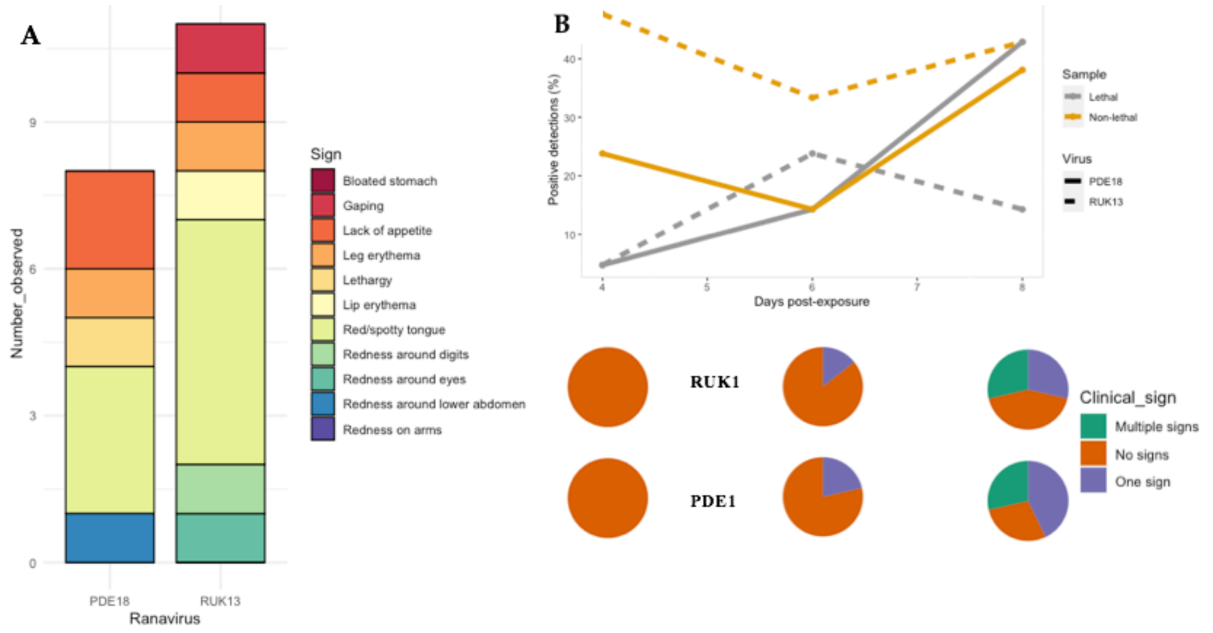

Figure S4. Clinical signs observed in *B.bufo* for both ranavirus treatment groups; a) in total, and b) by individual on sampling days compared to detectability of ranaviruses in non-lethal vs lethal samples.
